# ﻿*Rubustingzhouensis* (Rosaceae), a new species from Fujian, China

**DOI:** 10.3897/phytokeys.249.138951

**Published:** 2024-12-02

**Authors:** Ming Chen, Gui-Can Lin, Tao Wang, Yi-Xue Zhuang, Yi-Xin Yao, Cheng-Zi Yang, Yuan Qin, Yan-Xiang Lin, Chang An

**Affiliations:** 1 Fujian Maternity and Child Health Hospital, College of Clinical Medicine for Obstetrics & Gynecology and Pediatrics, Fujian Medical University, Fuzhou 350001, China Fujian Medical University Fuzhou China; 2 College of Pharmacy, Fujian University of Traditional Chinese Medicine, Fuzhou 350122, China Fujian University of Traditional Chinese Medicine Fuzhou China; 3 Fujian Health College, Fuzhou, Fujian 350101, China Fujian Health College Fujian China; 4 Xiamen Gaoxin School, Xiamen 361000, China Xiamen Gaoxin School Xiamen China; 5 State Key Laboratory of Quality Research in Chinese Medicine Institute of Chinese Medical Sciences, University of Macau, Macau 519000, China University of Macau Macau China; 6 Fujian Provincial Key Laboratory of Haixia Applied Plant Systems Biology, Center for Genomics and Biotechnology, College of Life Science, Fujian Agriculture and Forestry University, Fuzhou 350002, China Fujian Agriculture and Forestry University Fuzhou China

**Keywords:** Biodiversity, classification, floristic survey, morphology, new taxon

## Abstract

*Rubustingzhouensis* C.An & G.C.Lin, a newly-defined species within the family Rosaceae from Fujian Province, China, is formally described and illustrated here. *R.tingzhouensis* is morphologically similar to *R.swinhoei*, but can be distinguished by several key characteristics, such as long, reddish-purple stipitate glands, soft bristles, light yellow short trichomes (vs. shortly grey tomentose at the early stage, glabrescent) and scattered epidermal prickles (vs. few to many curved prickles). Furthermore, the stipules are pinnately deeply laciniate, measuring 1–1.5 cm (vs. ovate-oblong to ovate-lanceolate, 0.5–0.8 cm). Phylogenetic analyses, based on partial sequences and the complete plastome data, provide robust support for a close relationship between *R.tingzhouensis* and *R.swinhoei*, while also highlighting distinct genetic differentiation between these two species. The chloroplast genome of *R.tingzhouensis* is 156,311 bp in length and comprises 132 unique genes, including 86 protein-coding genes, 37 transfer RNAs, eight ribosomal RNAs and one pseudogene.

## ﻿Introduction

The genus *Rubus* L. (Rosaceae), established by Carl Linnaeus in 1753, initially comprised only ten species. Over the course of more than 270 years of botanical exploration and taxonomic refinement, *Rubus* has expanded to compass nearly 700 species (Gao et al. 2023; Huang et al. 2023). This expansion has made it one of the most speciose genera amongst angiosperms. This genus exhibits a broad distribution, predominantly spanning temperate and subtropical regions of the Northern Hemisphere. In China alone, over 208 species were documented, of which 139 are considered endemic (Lu and Boufford 2003). The high number of endemic species highlights China’s unique biodiversity and emphasises the importance of conservation efforts to protect these species, which are often vulnerable to habitat loss and other environmental pressures.

*Rubus* is distinguished by its diverse morphological characteristics, which include variations in leaf morphology, inflorescence structure, reproductive patterns, fruit colour and size, stem armature and other distinguishing traits (Woznicki et al. 2016; Xiong et al. 2019). The plants are typically characterised by the presence of bristles, prickles or glandular hairs and their leaves can be simple, palmate or pinnate. The floral structures are generally pentamerous and predominantly bisexual. The achenes are aggregated drupelets or drupaceous fruits borne on a peduncle, with fruit shapes varying from semi-spherical to conical or cylindrical (Yu et al. 2022).

The taxonomy of *Rubus* remains particularly challenging due to the extensive morphological variations exhibited by species within the genus. This complexity is further exacerbated by apomixis, polyploidy and frequent hybridisation events (Thompson 1997; Alice and Campbell 1999). Given its considerable significance in ornamental and medicinal value, there has been a growing interest amongst Chinese botanists in exploring the taxonomy of *Rubus*, including the description of new species and the identification of novel geographical distributions.

Plastome-based phylogenetic inference has emerged as a robust method for resolving relationships and identifying lineages within the family Rosaceae. This approach has been particularly effective in elucidating both shallow and deep phylogenies, as evidenced by several recent studies (Zhang et al. 2017; Liu et al. 2019; Liu et al. 2020a, 2020b; Liu et al. 2021; Su et al. 2021; Liu et al. 2022; Jin et al. 2023; Liu et al. 2023a, 2023b; Xu et al. 2023; Jin et al. 2024a, 2024b; Wang et al. 2024; Xue et al. 2024). These studies indicated that the plastome data could provide high-resolution insights into species boundaries, divergence times and the historical biogeography of the family, contributing significantly to the current understanding of both inter- and intra-generic relationships. In this study, we performed next-generation sequencing (NGS) for this potential new species and assembled the whole plastome to estimate its phylogenetic position in *Rubus*.

An unusual *Rubus* population was collected during a field expedition in April 2023 in Xuancheng Town, Changting County, Fujian Province, China. These plants had ovate to oblong-lanceolate leaves and racemose inflorescences, either terminal or axillary, similar to *R.swinhoei* Hance. However, they are distinct due to the dense covering of long, reddish-purple stipitate glands, soft bristles and short, light yellow trichomes, interspersed with scattered epidermal prickles on the stems. To clarify the taxonomic status of this population, we carried out molecular analyses to reconstruct phylogenetic trees involving 46 *Rubus* species. This approach provided insights into their phylogenetic relationships. The results confirmed that the collected population represents a new species. By comparing morphological and molecular evidence, we concluded that this species is indeed new and we provide its formal description and illustration here.

## ﻿Material and methods

### ﻿Morphological observations

We conducted extensive field surveys and detailed observation studies to assess the population status and phenological traits of the species. Specimens were collected during peak flowering periods, allowing for precise measurements of leaves, inflorescence and fruit structures, including their dimensions, features and colours. High-resolution photographs documented key characteristics and floral structures of the fully opened flowers were examined using a Leica M205a microscope. All specimens were deposited in the South China Botanical Garden Herbarium, Chinese Academy of Sciences (IBSC) and the China National Herbarium (PE) at the Institute of Botany, Chinese Academy of Sciences.

### ﻿DNA extraction and sequencing

Whole genomic DNA was extracted from fresh leaves using the DNeasy Plant Mini Kit (Qiagen, Valencia, CA, USA) and shipped to Jisi Huiyuan Biotechnology Co., Ltd. (Nanjing, Jiangsu) for sequencing. Quality assessment involved checking DNA integrity and concentration via gel electrophoresis and spectrophotometry. DNA was then fragmented by ultrasonication, purified and subjected to end repair, 3′ end adenylation, adapter ligation and gel electrophoresis for size selection. PCR amplification was used to generate sequencing libraries and only high-quality libraries were selected for paired-end (PE) sequencing on the Illumina NovaSeq 6000 platform, with a read length of 150 bp.

### ﻿Genome assembly, annotation and analysis

The sequencing data generated 19.82 GB of raw data. Bowtie2 v.2.2.4 (Langmead and Salzberg 2012) was used to align the reads against a custom chloroplast genome database for assembly. Annotation was performed using two complementary methods to enhance accuracy, verify results and improve reliability. Prodigal (https://github.com/hyattpd/Prodigal) was used for annotating chloroplast coding sequences (CDS), while hmmer v.3.4 and aragorn v.1.2.36 were used for predicting rRNA and tRNA genes (Laslett and Canback 2004; Potter et al. 2018). Additionally, gene sequences from related species available on NCBI were aligned against the assembled sequences using BLAST v.2.6 (https://blast.ncbi.nlm.nih.gov/Blast.cgi) for alternative annotations. A comparative analysis between the two annotation sets was conducted and discrepancies were resolved manually to ensure accuracy and eliminate redundancies. Finally, OrganellarGenomeDRAW (OGDRAW) was used to generate the chloroplast genome map (Greiner et al. 2019).

### ﻿Phylogenetic study

To determine the phylogenetic position of the new species within *Rubus*, chloroplast genome sequences of 48 species, including *Rubus* species, were retrieved from the NCBI database (Suppl. material 2), with *Rosalaevigata* Michx. and *Pygeumtopengii* Merr. serving as outgroups. The sequences were aligned using MAFFT v.7.310 with default parameters (Katoh and Standley 2013) and Maximum Likelihood phylogenetic inference was performed with RAxML-NG (Kozlov et al. 2019) using the GTRGAMMA model, with a rapid Bootstrap analysis of 1000 replicates. The resulting phylogenetic tree was visualised using the ChiPlot online programme (Xie et al. 2023).

## ﻿Results

### ﻿Morphological characteristics of *Rubustingzhouensis*

Specimens of *Rubustingzhouensis* were compared with type specimens of several closely-related species within *Rubus*, revealing significant morphological differences, particularly in leaf (e.g. unique shape patterns), indumentum texture (e.g. density and colour of trichomes) and stipules (e.g. shape and size) (Suppl. material 1). To provide a comprehensive description, we measured and documented features such as branchlets, leaves, and floral structures, including indumentum, prickles, stipules and inflorescences. These morphological traits were compared with those of *R.tingzhouensis*, *R.swinhoei* and *R.amphidasys* Focke (Table 1). Notable differences in the indumentum, as well as distinct variations in stipules and stamens, strongly support the classification of *R.tingzhouensis* as a new species.

**Table 1.** Morphological comparison between *R.tingzhouensis* and its allied species.

Differentiating characters		*Rubusamphidasys*	*Rubusswinhoei*	*Rubustingzhouensis*
Habit		trailing shrubs	climbing shrubs	climbing shrubs
Branchlets		Reddish-brown	brown to purplish-brown	Reddish-brown
Indumentum		dense reddish-brown long stipitate glands, soft bristles and long yellowish hairs	shortly grey tomentose at first, glabrescent	densely covered with reddish-purple long stipitate glands, soft bristles and light-yellow short hairs, with scattered epidermal prickles
Prickles		usually unarmed	with few to many curved prickles	sparsely prickles
Stipules	shape & size	deeply laciniate, 0.8–1.5 cm	ovate-oblong to ovate-lanceolate,0.5–0.8 cm	deeply laciniate, 1–1.5 cm
	indumentum	with long glandular hairs, villous		with long glandular hairs, villous
Leaves	petioles	20–55 mm	5–10(15) mm	15–20 mm
	shape	broadly to narrowly ovate	ovate to oblong-lanceolate,	ovate to oblong-lanceolate,
	size	5–11 × 3.5–9 cm	5–11 × 2.5–5 cm	8–16 × 3.5–6 cm
	indumentum	both surfaces villous	adaxially glabrous, abaxially grey tomentose, or subglabrous	adaxially hirsutullous, purple long stipitate glands along veins, abaxially densely yellowish-brown tomentose and pubescent
	base, margin and apex	base cordate, margin 3–5-lobed, terminal lobe much larger and longer than lateral lobes, apex shortly acuminate, rarely acute, lateral lobes obtuse, unevenly sharply serrate.	base rounded or truncate to shallowly cordate, margin unevenly serrate to doubly serrate, rarely incised, apex acuminate.	base cordate, margin unevenly serrate to doubly serrate, apex acuminate to acute.
Inflorescences	position	terminal or axillary	terminal	terminal or axillary
Flower	number and size	5–12, 1–1.5 cm	5–6, 1–1.5 cm	5–10, 2–2.5 cm
	corolla	Petals white, broadly ovate to oblong, 4–7 × 3–5 mm, base barely clawed, margin premorse or coarsely serrate	Petals white, broadly obovate to suborbicular, 5–7 × 4–6 mm, both surfaces thinly pubescent, base shortly clawed, apex obtuse, undulate.	Petals white, broadly ovate to oblong, 5–6 × 4–5 mm, base barely clawed, apex slightly concave.

*: *R.amphidasys* exhibits significant differences in leaf morphology and growth form compared to the newly-described species; however, it possesses similar indumentum characteristics, warranting its inclusion in the comparative analysis.

### ﻿Phylogenetic analysis

**Figure 1.**
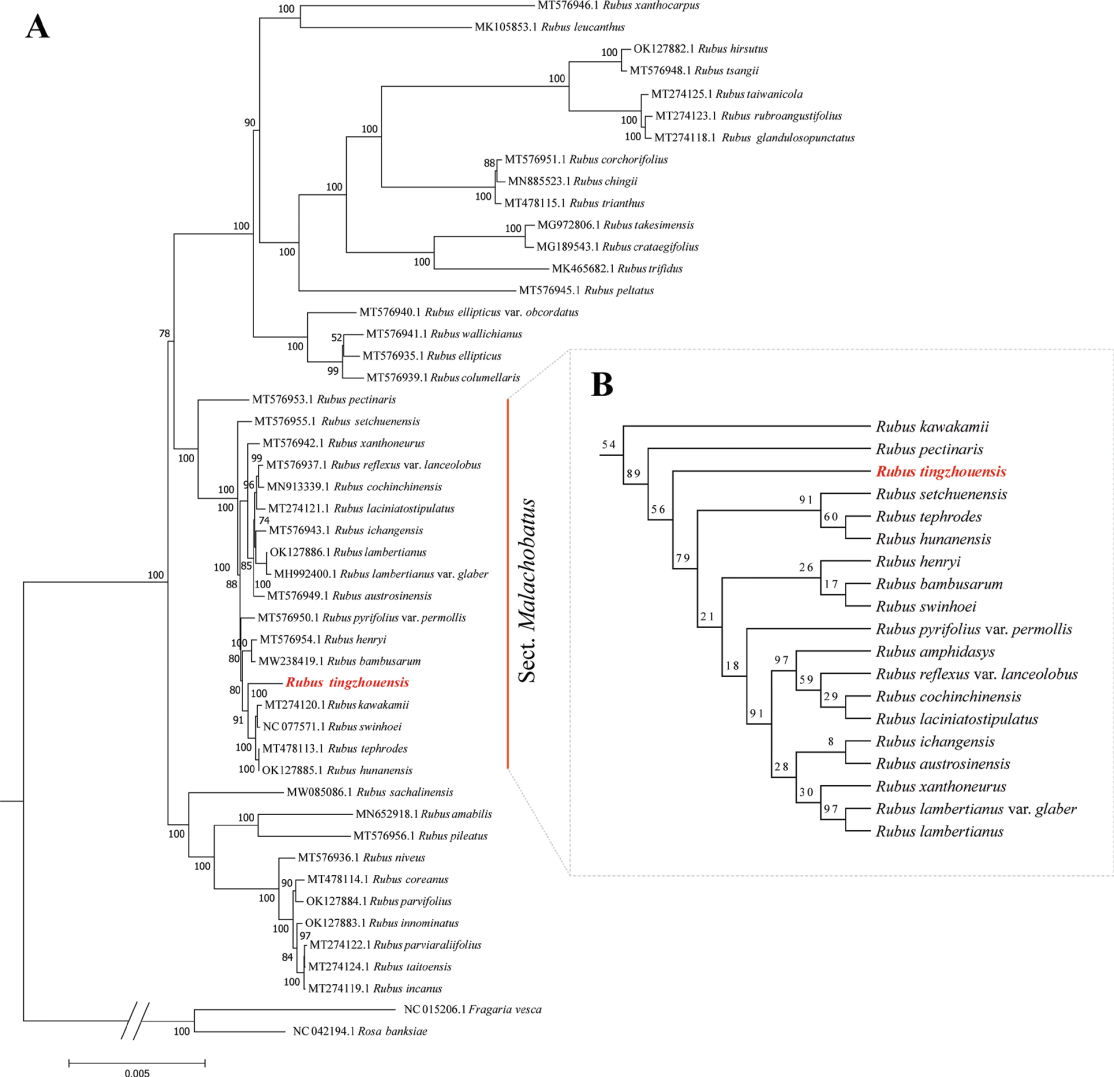
Phylogenetic trees of *Rubus* reconstructed separately based on (A) complete plastome DNA sequences and (B) plastid fragments (*rbc*L, r pl20-*rps*12 and *trn*G-*trn*S). Numbers at nodes represent Maximum Likelihood bootstrap percentages (BP). The new species sequenced in this study are highlighted in red font.

Building on previous molecular studies (Wang et al. 2016; Xu et al. 2024), our dataset included 46 *Rubus* species and two outgroup species. Phylogenetic analysis of complete and partial chloroplast genome sequences confirmed that *R.tingzhouensis* represents a distinct species, supported by a high bootstrap value and unique clade positioning within sect. Malachobatus (Fig. 1). The chloroplast genome tree positioned *R.tingzhouensis* within the sect. Malachobatus Focke, forming a clade with four other members of this section, with a strong bootstrap value (BP = 91). Specifically, *R.tephrodes* Hance and *R.hunanensis* Hand.-Mazz. formed a sister group, consistent with previous findings (Yu et al. 2022), while *R.kawakamii* Hayata and *R.swinhoei* exhibited a closer phylogenetic relationship. Within this clade, *R.tingzhouensis* forms a distinct lineage.

### ﻿The chloroplast genome features of *Rubuschangii*

**Figure 2.**
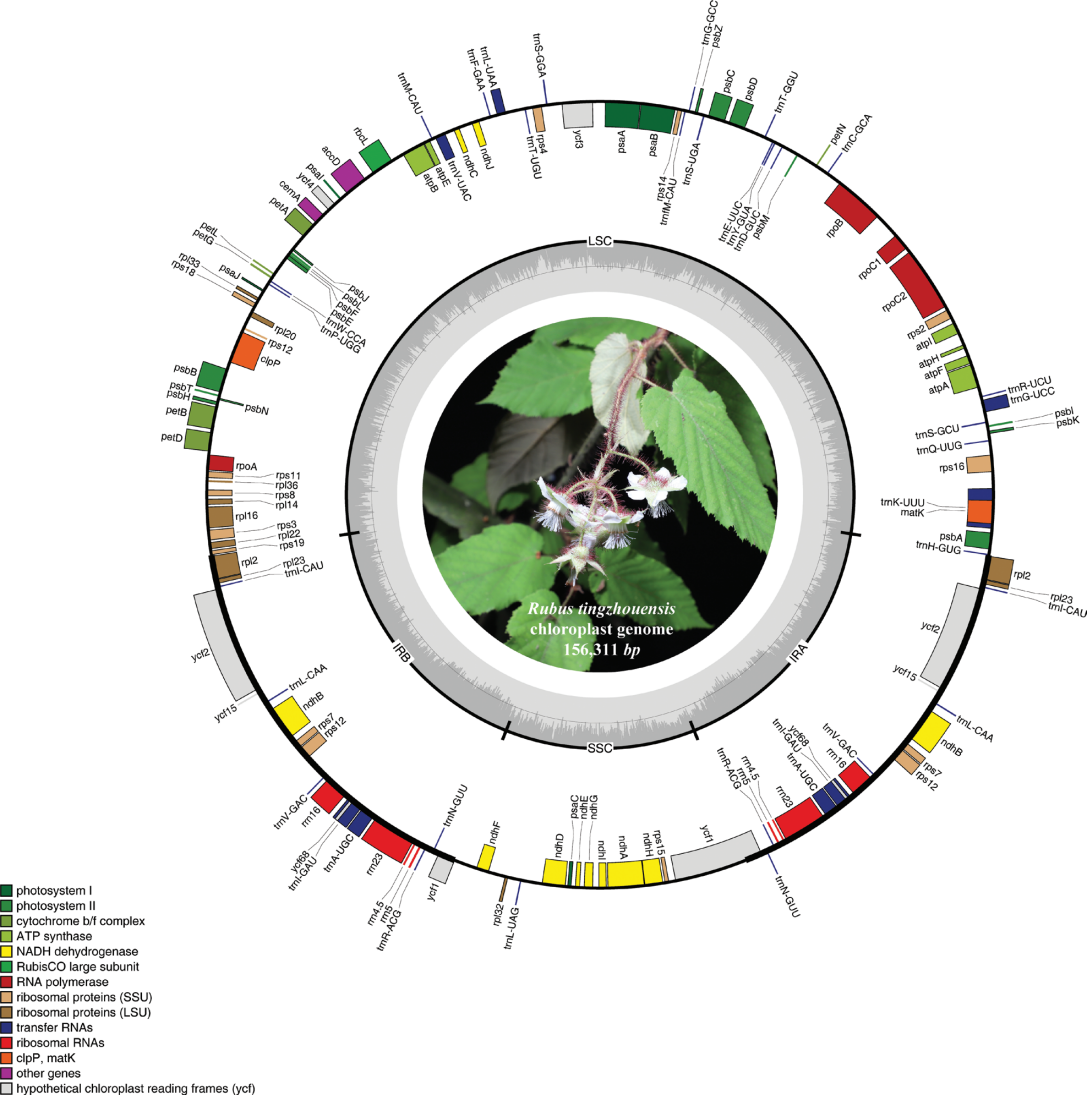
Gene map of the *R.tingzhouensis* plastome. Two purple scalloped areas identify the inverted repeat regions (IRa and IRb). Genes drawn inside and outside of the map are transcribed in clockwise and counter-clockwise directions, respectively. The dark grey bar graphs inner circle shows the GC content.

The complete annotated chloroplast genome of *R.tingzhouensis* is a double-stranded circular DNA with a length of 156,311 bp (Fig. 2). This genome length is comparable to that of other species within the Rubus genus, indicating a conserved genomic structure typical of the family Rosaceae. The chloroplast genome consists of two inverted repeats (IR) regions (IRA and IRB, each 25,801 bp), a large single-copy (LSC) region (85,842 bp) and a small single-copy (SSC) region (18,867 bp). The LSC, SSC and IR regions account for 55.4%, 12.3% and 32.3% of the total length, respectively. These proportions are typical for chloroplast genomes in *Rubus* and contribute to genome stability and gene expression regulation, consistent with related species in the Rosaceae family. The overall GC content is 37.18%. The *R.tingzhouensis* chloroplast genome contains 132 unique genes, including 86 protein-coding genes, 37 transfer RNAs, eight ribosomal RNAs and one pseudogene.

Introns are present in several coding genes, which is consistent with other chloroplast genomes of flowering plants. Of the 86 protein-coding genes, 16 contain introns (Table 2). Two genes, *clp*P and *rps*12 have two introns each, while 12 additional genes (*ndh*A, *ndh*B, *pet*B, *pet*D, *rpl*16, *rpl*2, *rps*16, *trn*A-UGC, *trn*G-UCC, *trn*I-GAU, *trn*K-UUU, *trn*L-UAA and *trn*V-UAC) contain a single intron each.

**Table 2.** Gene composition in the chloroplast genome of *R.tingzhouensis*.

Category	Gene group	Gene name
Photosynthesis	Subunits of photosystem I	*psaA*, *psaB*, *psaC*, *psaI*, *psaJ*
	Subunits of photosystem II	*psbA*, *psbB*, *psbC*, *psbD*, *psbE*, *psbF*, *psbH*, *psbI*, *psbJ*, *psbK*, *psbL*, *psbM*, *psbN*, *psbT*, *psbZ*
	Subunits of NADH dehydrogenase	ndhA*, ndhB*(2), *ndhC*, *ndhD*, *ndhE*, *ndhF*, *ndhG*, *ndhH*, *ndhI*, *ndhJ*
	Subunits of cytochrome b/f complex	*petA*, petB*, petD*, *petG*, *petL*, *petN*
	Subunits of ATP synthase	*atpA*, *atpB*, *atpE*, *atpF*, *atpH*, *atpI*
	Large subunit of rubisco	*rbcL*
	Subunits photochlorophyllide reductase	–
Self-replication	Proteins of large ribosomal subunit	*rpl14*, rpl16*, rpl2*(2), *rpl20*, *rpl22*, *rpl23(2)*, *rpl32*, *rpl33*, *rpl36*
	Proteins of small ribosomal subunit	*rps11*, rps12**(2), *rps14*, *rps15*, rps16*, *rps18*, *rps19*, *rps2*, *rps3*, *rps4*, *rps7(2)*, *rps8*
	Subunits of RNA polymerase	*rpoA*, *rpoB*, *rpoC1*, *rpoC2*
	Ribosomal RNAs	*rrn16(2)*, *rrn23(2)*, *rrn4.5(2)*, *rrn5(2)*
	Transfer RNAs	trnA-UGC*(2), *trnC-GCA*, *trnD-GUC*, *trnE-UUC*, *trnF-GAA*, *trnG-GCC*, trnG-UCC*, *trnH-GUG*, *trnI-CAU(2)*, trnI-GAU*(2), trnK-UUU*, *trnL-CAA(2)*, trnL-UAA*, *trnL-UAG*, *trnM-CAU*, *trnN-GUU(2)*, *trnP-UGG*, *trnQ-UUG*, *trnR-ACG(2)*, *trnR-UCU*, *trnS-GCU*, *trnS-GGA*, *trnS-UGA*, *trnT-GGU*, *trnT-UGU*, *trnV-GAC(2)*, trnV-UAC*, *trnW-CCA*, *trnY-GUA*, *trnfM-CAU*
Other genes	Maturase	*matK*
	Protease	clpP**
	Envelope membrane protein	*cemA*
	Acetyl-CoA carboxylase	*accD*
	c-type cytochrome synthesis gene	–
	Translation initiation factor	–
	other	–
Genes of unknown function	Conserved hypothetical chloroplast ORF	#*ycf1*, *ycf1*, *ycf15(2)*, *ycf2(2)*, ycf3**, *ycf4*, *ycf68(2)*

Gene*: Gene with one intron; Gene**: Gene with two introns; Gene(2): Number of copies of multi-copy genes.

### ﻿Taxonomic treatment

#### ﻿ Rubus tingzhouensis

Taxon classificationPlantaeRosalesRosaceae

C.An & G.C.Lin sp. nov.

7E0D929D-4AEA-576B-B1D9-44A98979533A

urn:lsid:ipni.org:names:77352705-1

Figs 3 , 4

##### Type.

China • Fujian: Longyan City, Changting County, Xuancheng Town, Xiashe Village, 25°24'06"N, 116°22'34"E, forests on mountain slopes, alt. ca. 351 m, 18 April 2024, *C. An & G.C. Lin. 240418* (holotype: IBSC [barcode 1021457!]; isotypes: PE [barcode 02468523!, 02468524!]).

**Figure 3.**
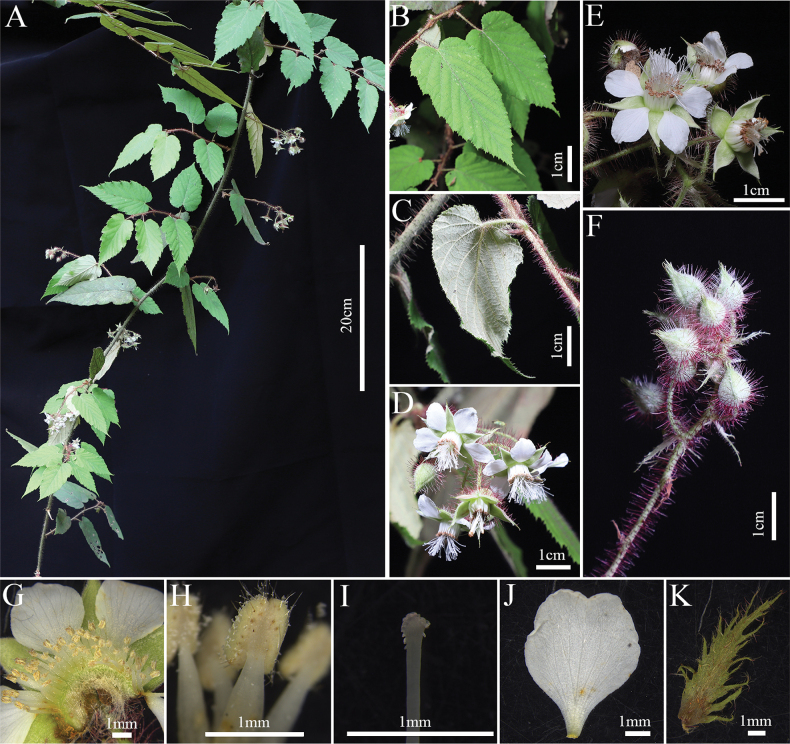
*Rubustingzhouensis* C. An & G. C. Lin **A** plant in natural habitat **B** leaf (adaxial surface) **C** leaf (abaxial surface) **D, E, F** flower and inflorescence **G** androecium **H** stamen **I** stigma **J** petal **K** stipule.

##### Diagnosis.

This species is similar to *R.swinhoei* in its growth habit, with ovate to oblong-lanceolate leaf blades and botryoid inflorescences that may be terminal or axillary. However, *R.tingzhouensis* can be distinguished by its dense indumentum of long, reddish-purple stipitate glands, soft setae and light yellow short trichomes on the plant surface. It also has scattered epidermal prickles, adding to its distinct appearance. Additionally, it has deeply laciniate stipules measuring 1–1.5 cm in length, which are significantly more divided than those of related species, making them a key distinguishing feature.

**Figure 4.**
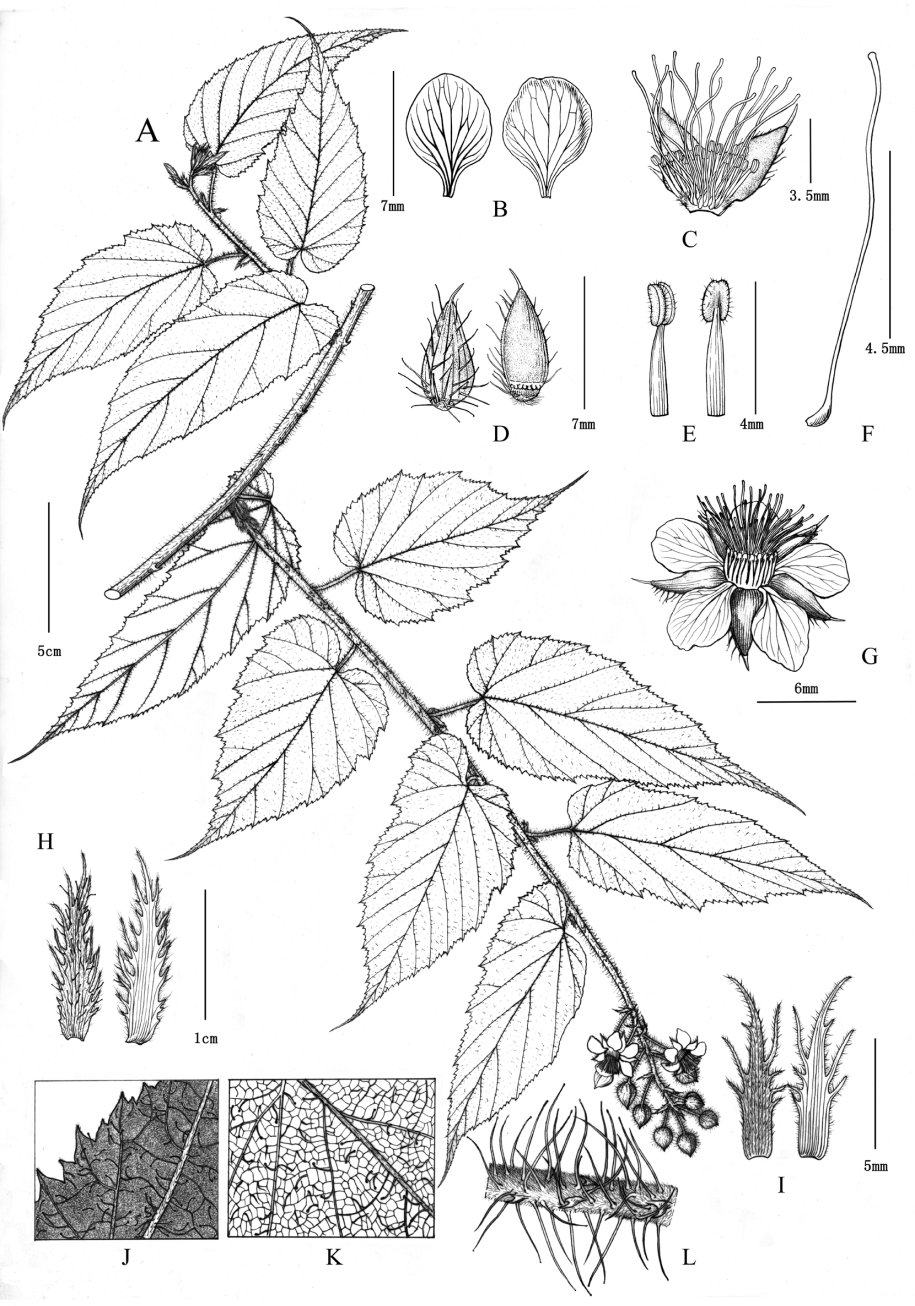
Illustration of *Rubustingzhouensis* C. An & G. C. Lin **A** habit **B** petal **C** longitudinal section of flower **D** free lobes of calyx **E** stamen **F** pistil **G** flower **H** stipule **I** bract **J** margin and trichome of leaf **K** trichome of abaxial leaf **L** section of leafed stem. Drawn by Yunxiao Liu.

##### Description.

**Vines, lianas and shrubs. *Stems*** cylindrical, greyish-brown, with dense, reddish-purple long glandular hairs, soft bristles, short yellowish hairs and sparse prickles, apically rooting. ***Leaves*** simple; blades ovate to oblong-lanceolate, herbaceous, 8–16 × 3.5–6 cm, apex acuminate to acute, base cordate; adaxially flat, hirsutullous with long, purple stipitate glands along veins, abaxially densely yellowish-brown tomentose and pubescent, with long soft hairs along mid-ribs; principal veins sparsely retrorsely aculeolate, margin unevenly serrate to doubly serrate, apex acuminate to acute, lateral veins 9 to 10 pairs; petiole 1.5–2 cm, with dense, long, purplish-red glandular hairs and soft bristles; stipules caducous, free, pinnatipartite, lobes narrowly elliptic or lanceolate, densely covered with long glandular hairs and tomentose-villous, 1–1.5 cm. ***Inflorescences*** terminal or axillary, short botryoid, 5–10 flowered; involucral bracts 6–9 mm, lobed, lobes linear or lanceolate, villous, rachis and pedicels with dense reddish-purple long glandular hairs and soft bristles; peduncle 5–10 cm, pedicels 1.5–2 cm; ***Flowers*** 2–2.5 cm. Sepals ovate-lanceolate, 5–6 mm, apex acuminate to caudate, outer sepals usually 2- or 3-laciniate; abaxially densely greyish-white pubescent, purplish-red long glandular hairs and soft bristles, adaxially densely greyish-white pubescent. Petals white, broadly ovate to oblong, 5–6 × 4–5 mm, base barely clawed, slightly shorter than sepals. Stamens many; filaments linear, lower part slightly broader; anthers with few long hairs. Carpels many, style longer than stamens, glabrous.

##### Phenology.

Flowering in March to May, fruits have not been seen yet.

##### Etymology.

The specific epithet ‘tingzhouensis’ refers to the ancient region Tingzhou (汀州) in south-western Fujian, China, where this species was discovered. The Chinese name, “红毛木莓” (hong mao mu mei), reflects the plant’s dense covering of reddish-purple long hairs.

##### Distribution and ecology.

**Figure 5.**
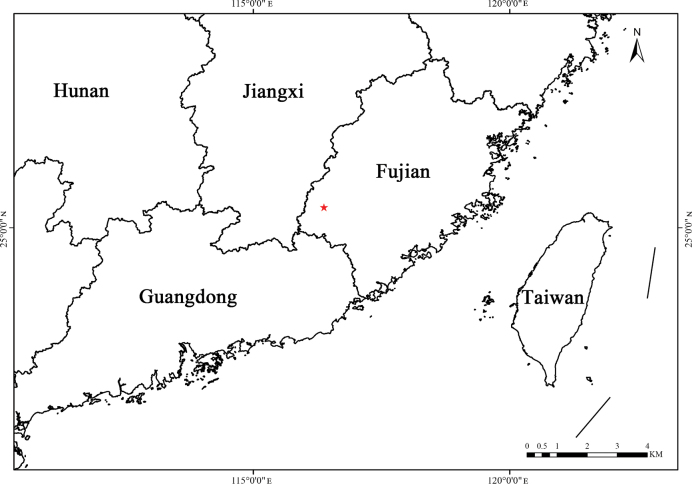
Geographical distribution of *R.tingzhouensis* in China (red star).

Currently, this species is only found in Changting County and Shanghang County, Fujian Province, China (Fig. 5). It is sporadically distributed in the understorey of the primary forest in mountain valleys at an altitude of 300–400 m. The habitat features a thick layer of dead branches and leaf litter, as well as a substantial amount of humus, supporting vigorous growth of herbaceous, shrubby and woody plants. Associated species include *Verniciamontana* Lour. and *Pterocaryastenoptera* C.D.C. in the tree layer; *R.corchorifolius* L.f., *Buddlejalindleyana* Fortune, *Diplosporadubia* (Lindl.) Masam., *Callicarpakochiana* Makino, *Ilexpubescens* Hook. & Arn., *Iteaomeiensis* C.K.Schneid., *C.formosana* R.Br., *Clerodendrumcyrtophyllum* Turcz., *Loropetalumchinense* (R.Br.) Oliv., *Mallotusapelta* (Lour.) Müll.Arg., *Trematomentosa* (Roxb.) H.Hara, *Phyllanthusglaucus* Wall. ex Müll.Arg., *Melastomamalabathricum* L. and *R.reflexus* Ker Gawl. in the shrub layer; *Lysimachiaalfredii* Hance, *Senecioscandens* Buch.-Ham. ex D.Don, *Blechnopsisorientalis* (L.) C.Presl and *Dicranopterispedata* (Houtt.) Nakaike in the herbaceous layer.

##### Conservation assessment.

*R.tingzhouensis* is documented in limited populations distributed within sparse forests on mountain slopes in Shanghang, Changting and Wuping County, Fujian Province or thrives along stream margins and under mixed forests. Notably, one population in Changting County is adjacent to a scenic locale, heightening its susceptibility to considerable anthropogenic disturbance. Furthermore, most of these populations are located outside designated conservation zones, making them vulnerable to ongoing exploitation of woodland resources by local residents. Thus, this newly-recognised species is assigned a preliminary status of Vulnerable (VU D2) according to the IUCN Red List Categories and Criteria (Standards and Group 2006; Betts et al. 2020), reflecting a population with a severely limited occupancy range (typically less than 20 km^2^) or few locations (typically five or fewer).

## ﻿Discussion

Based on the well-supported phylogenetic trees (Fig. 2), *Rubustingzhouensis* is identified as a member of Sect. Malachobatus Focke. This species shares a close relationship with *R.swinhoei*, which aligns strongly with our morphological observations. Combining its distinct morphological differences with the high-support phylogenetic relationship, we confirm its designation as a new species. Despite its morphological resemblance to *R.swinhoei*, this new species exhibits distinct differences in pubescence and stipule morphology; for instance, it is densely covered with long, reddish-brown stipitate glands, soft bristles and light-yellow short hairs, with scattered epidermal prickles and its stipules are pinnately deeply laciniate. These disparities robustly substantiate its status as a new species, a conclusion further supported by subsequent phylogenetic analyses. It is noteworthy that *R.swinhoei* is widely distributed globally. However, *R.tingzhouensis* is an endemic species found in the south-western mountainous regions of Fujian Province, typically occurring at altitudes around 300 m. Currently, the discovered population of this species is limited, warranting intensified efforts in population surveys and conservation endeavours.

Due to its straightforward and stable genetic architecture, coupled with its amenability to sequencing, the chloroplast genome has garnered increasing attention for applications in species identification, phylogenetic reconstruction, demographic history elucidation and species divergence investigations (Palmer 1987; Liu et al. 2020c). Nevertheless, genomic data pertaining to *Rubus*, especially complete chloroplast genomes within the NCBI database, remained notably scarce. In this present investigation, we conducted the sequencing and assembly of the entire chloroplast genome of the new *Rubus* species. Its structure resembles that of most angiosperms (Palmer and Stein 1986; Dobrogojski et al. 2020), comprising a circular double-stranded molecule spanning 156,311 bp in total length. In detail, it exhibits the characteristic quadripartite organisation consisting of a large single-copy region (LSC), a small single-copy region (SSC) and a pair of inverted repeat regions (IR), measuring 85,842, 18,867 and 25,801 bp, respectively. Furthermore, the GC content is 37.18%. This structure is consistent with the genomic organisation in reported *Rubus* species (Lu et al. 2024).

However, the plastid phylogeny only represented the maternally inherited phylogeny and cannot depict the accurate evolutionary history of *Rubus*. Looking ahead, the phylogenomic era, characterised by the analysis of hundreds or even thousands of single-copy nuclear genes (SCNs), is rapidly gaining momentum across the plant systematic community (Liu et al. 2021). The nuclear SCNs-based research promises to revolutionise our understanding of plant evolution, offering unprecedented insights into the genetic underpinnings of diverse plant groups and various fields (Jin et al. 2023; Xu et al. 2023). As we delve deeper into this new era, our study serves as a critical stepping-stone, providing a robust and comprehensive framework that will undoubtedly facilitate future research. By leveraging the power of plastome data and the emerging techniques in phylogenomic analysis, we are poised to uncover new layers of complexity and diversity within the plant kingdom, enriching our understanding of its evolutionary history.

### ﻿Additional specimens examined

***Rubusswinhoei* Hance.**—China • Taiwan, Tamsui; Apr. 1864 (fl.); holotype: Richard Oldham 03341152 (P) • Guangdong, Lo-Fau-Shan; 2600 m alt.; May 1883 (fl.); isotype: James Alexander Calder 000946959 (BM) • Taiwan, Nan-To and mountains northwards; Oct 1887 (fl.); holotype: A. Henry 000737663 (K); syn. ***R.hupehensis*** • Fujian, Kuatun; May 1898 (fl.); holotype: M. de Latouche 00755178 (P); syn. ***R.adenanthus*** • Taiwan, Shichiseitonzan; Mar 1911 (fl.); holotype: Yaichi Shimada 12172 (TAIF); syn. ***R.adenotrichopodus***.

***R.amphidasys* Focke ex Diels**—China • Sichuan, Mont du College, Gorges Yang-pa; aest. 1891 (fl.); holotype: Rosthorn 1172563 (B).

***R.doyonensis* Hand. - Mazz.**—China • Yunnan, near the village of Bahan (Pehalo), in warm, mixed rainforests by the Nu-jiang (Salween) River; 2600 m alt.; 20 Jun 1916 (fl.); holotype: Heinrich Handel-Mazzetti 0059386 (WU).

***R.hanceanus* Kuntze**—China • Guangdong, West River, near Foshing; 18 May 1882 (fl.); paratype: C. Ford 000737848 (K).

## Supplementary Material

XML Treatment for Rubus tingzhouensis

## ﻿Additional information

### Author ORCIDs

Ming Chen https://orcid.org/0009-0001-9894-8339

Gui-Can Lin https://orcid.org/0009-0001-7911-358X

Tao Wang https://orcid.org/0009-0001-5749-4174

Yi-Xue Zhuang https://orcid.org/0000-0002-0295-9405

Yi-Xin Yao https://orcid.org/0000-0001-6948-6975

Cheng-Zi Yang https://orcid.org/0000-0002-5527-7934

Yuan Qin https://orcid.org/0000-0003-4713-6151

Yan-Xiang Lin https://orcid.org/0009-0008-6971-792X

Chang An https://orcid.org/0000-0002-7657-7936

### Data availability

All of the data that support the findings of this study are available in the main text or Supplementary Information.

## Supplementary materials

Supplementary material 1 The type specimens of closely-related species of *Rubustingzhouensis* Data type png Explanation note **A** holotype of *R.swinhoei*, P03341152 **B** holotype of of *R.adenanthus*, P00755178 **C** holotype of *R.hupehensis*, K000737663 **D** holotype of *R.amphidasys*, B10 1172563 **E** holotype of *R.doyonensis*, WU0059386 **F** paratype of *R.hanceanus*, K000737848. https://binary.pensoft.net/file/1188400This dataset is made available under the Open Database License (http://opendatacommons.org/licenses/odbl/1.0/). The Open Database License (ODbL) is a license agreement intended to allow users to freely share, modify, and use this Dataset while maintaining this same freedom for others, provided that the original source and author(s) are credited.Chang An

Supplementary material 2 Taxa and GenBank accession numbers for plastome sequences used in this study Data type xls https://binary.pensoft.net/file/1188401This dataset is made available under the Open Database License (http://opendatacommons.org/licenses/odbl/1.0/). The Open Database License (ODbL) is a license agreement intended to allow users to freely share, modify, and use this Dataset while maintaining this same freedom for others, provided that the original source and author(s) are credited.Chang An

Supplementary material 3 Digital photos of plants *R.amphidasys* Data type zip https://binary.pensoft.net/file/1188402This dataset is made available under the Open Database License (http://opendatacommons.org/licenses/odbl/1.0/). The Open Database License (ODbL) is a license agreement intended to allow users to freely share, modify, and use this Dataset while maintaining this same freedom for others, provided that the original source and author(s) are credited.Chang An

Supplementary material 4 Digital photos of plants *R.swinhoei* Data type zip https://binary.pensoft.net/file/1188403This dataset is made available under the Open Database License (http://opendatacommons.org/licenses/odbl/1.0/). The Open Database License (ODbL) is a license agreement intended to allow users to freely share, modify, and use this Dataset while maintaining this same freedom for others, provided that the original source and author(s) are credited.Chang An

Supplementary material 5 Digital photos of plants *R.tingzhouensis* Data type zip https://binary.pensoft.net/file/1188404This dataset is made available under the Open Database License (http://opendatacommons.org/licenses/odbl/1.0/). The Open Database License (ODbL) is a license agreement intended to allow users to freely share, modify, and use this Dataset while maintaining this same freedom for others, provided that the original source and author(s) are credited.Chang An
