# Supplementary figures and images for: ﻿Rubustingzhouensis (Rosaceae), a new species from Fujian, China
Source: PhytoKeys. 2024 Dec 2;249:251–67. doi: 10.3897/phytokeys.249.138951 (PMC11629084; doi:10.3897/phytokeys.249.138951)

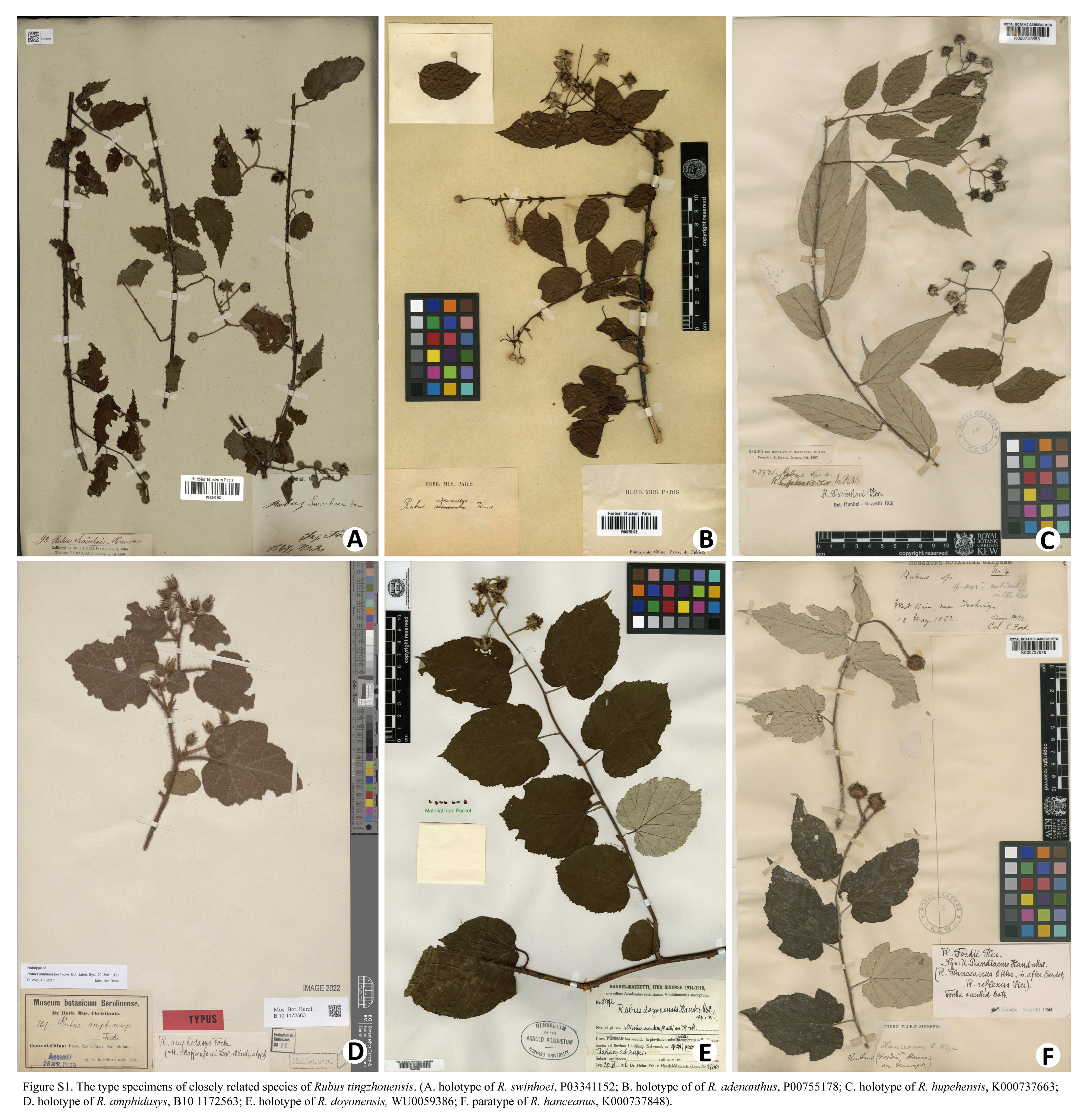

Supplement: Supplementary material 1 — The type specimens of closely-related species of Rubustingzhouensis [file phytokeys-249-251_article-138951__-s001.png]

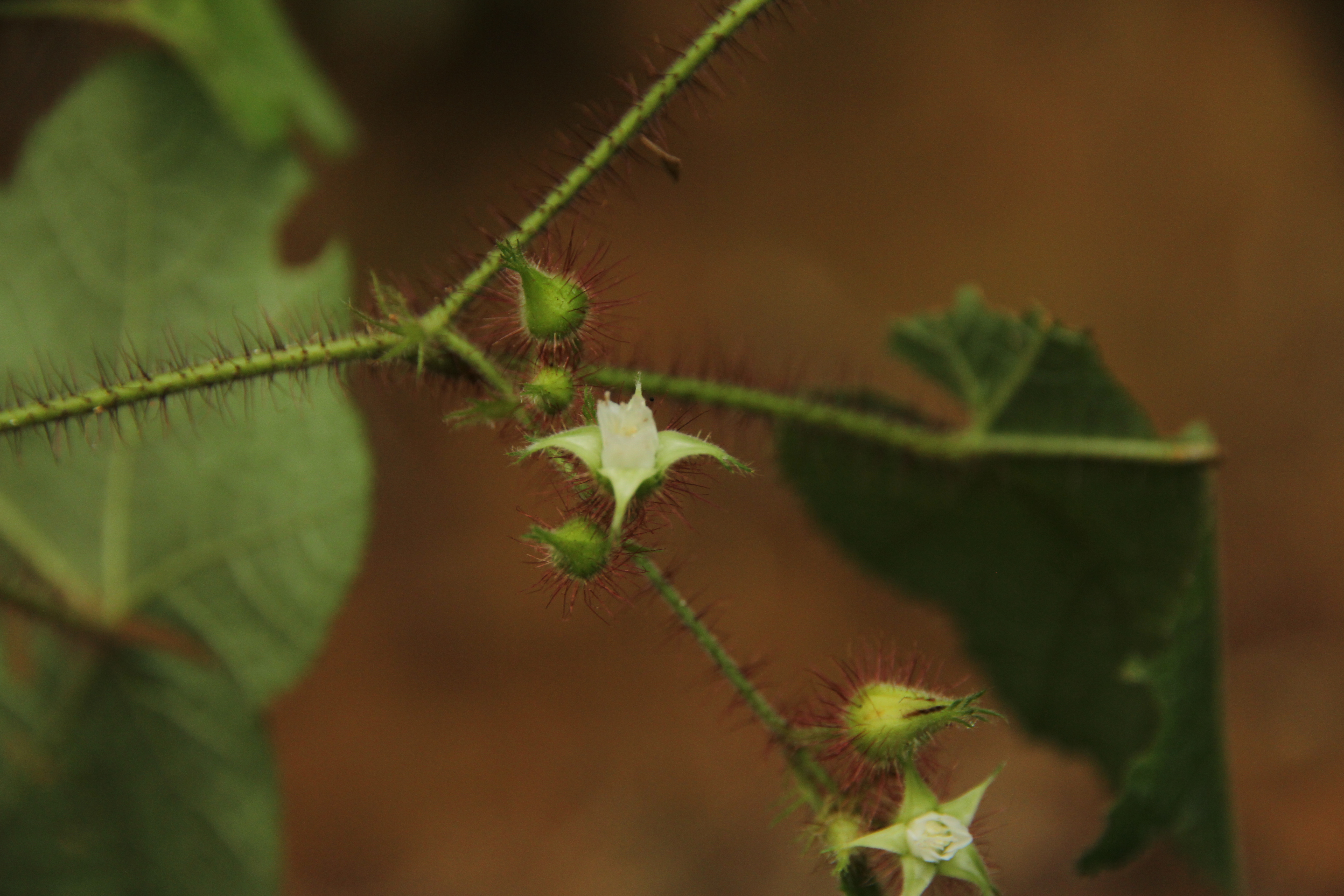

Supplement: Supplementary material 3 — Digital photos of plants R.amphidasys [file phytokeys-249-251_article-138951__-s003.zip › R_amphidasys/Rubus amphidasys_1.jpg]

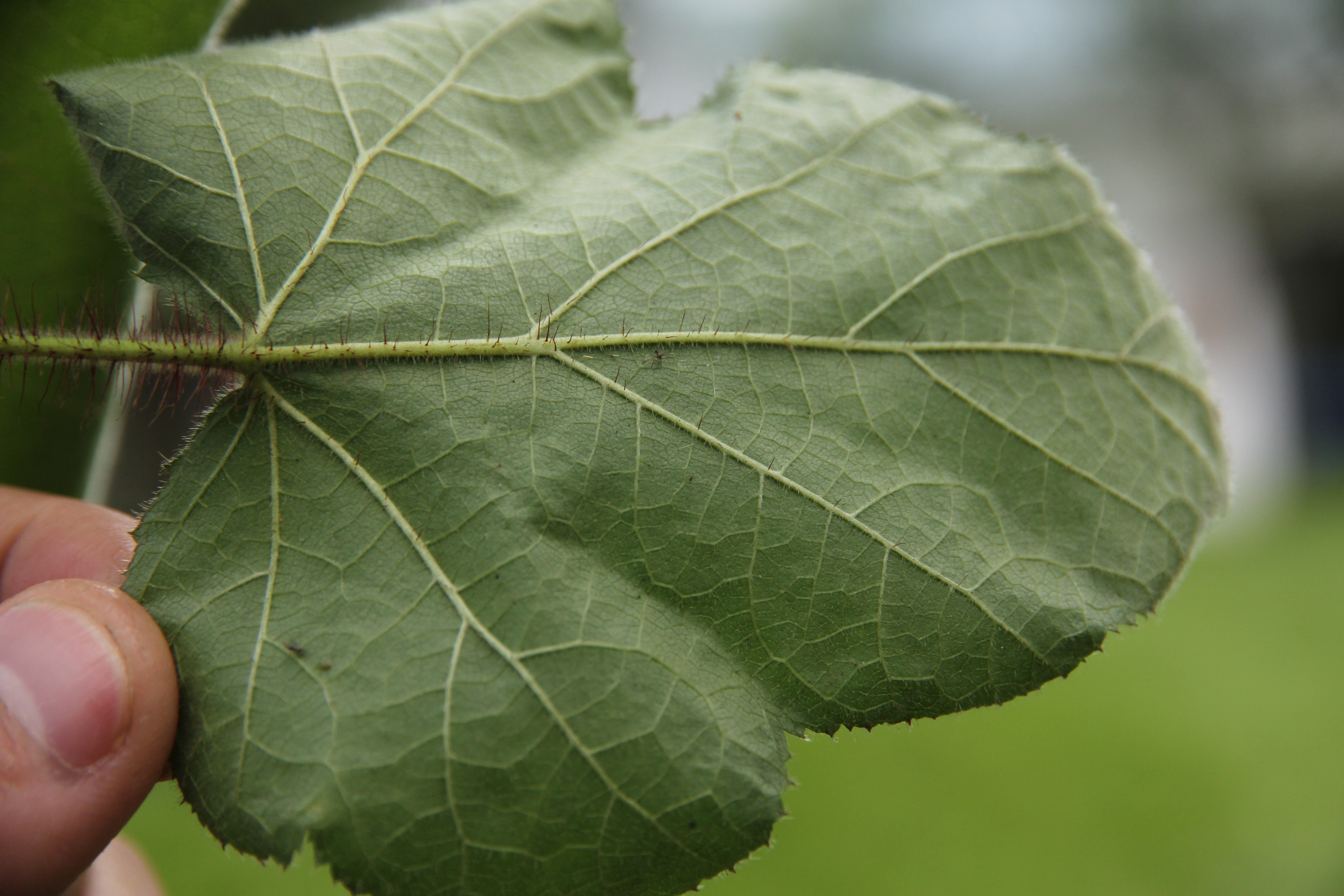

Supplement: Supplementary material 3 — Digital photos of plants R.amphidasys [file phytokeys-249-251_article-138951__-s003.zip › R_amphidasys/Rubus amphidasys_2.jpg]

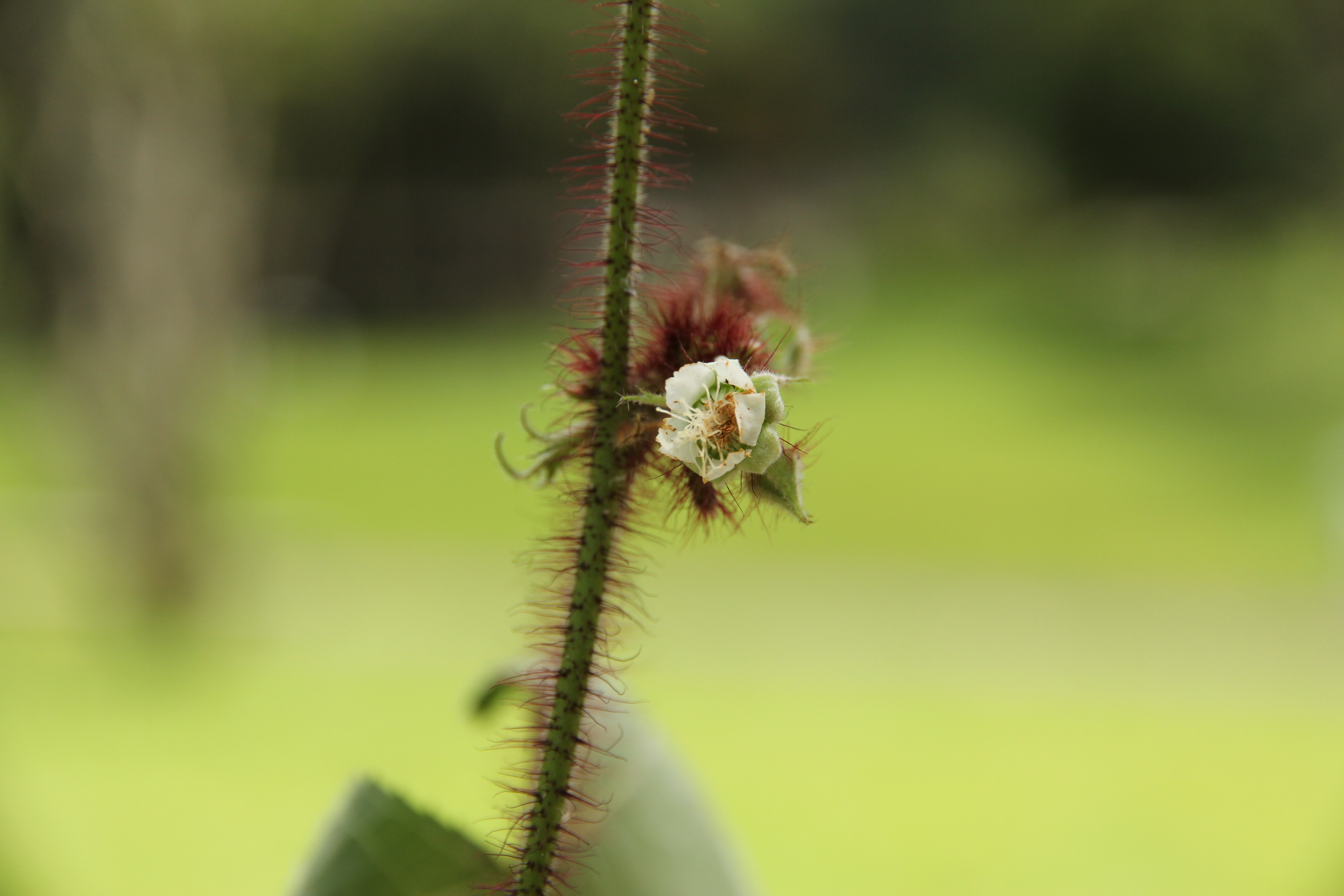

Supplement: Supplementary material 3 — Digital photos of plants R.amphidasys [file phytokeys-249-251_article-138951__-s003.zip › R_amphidasys/Rubus amphidasys_3.jpg]

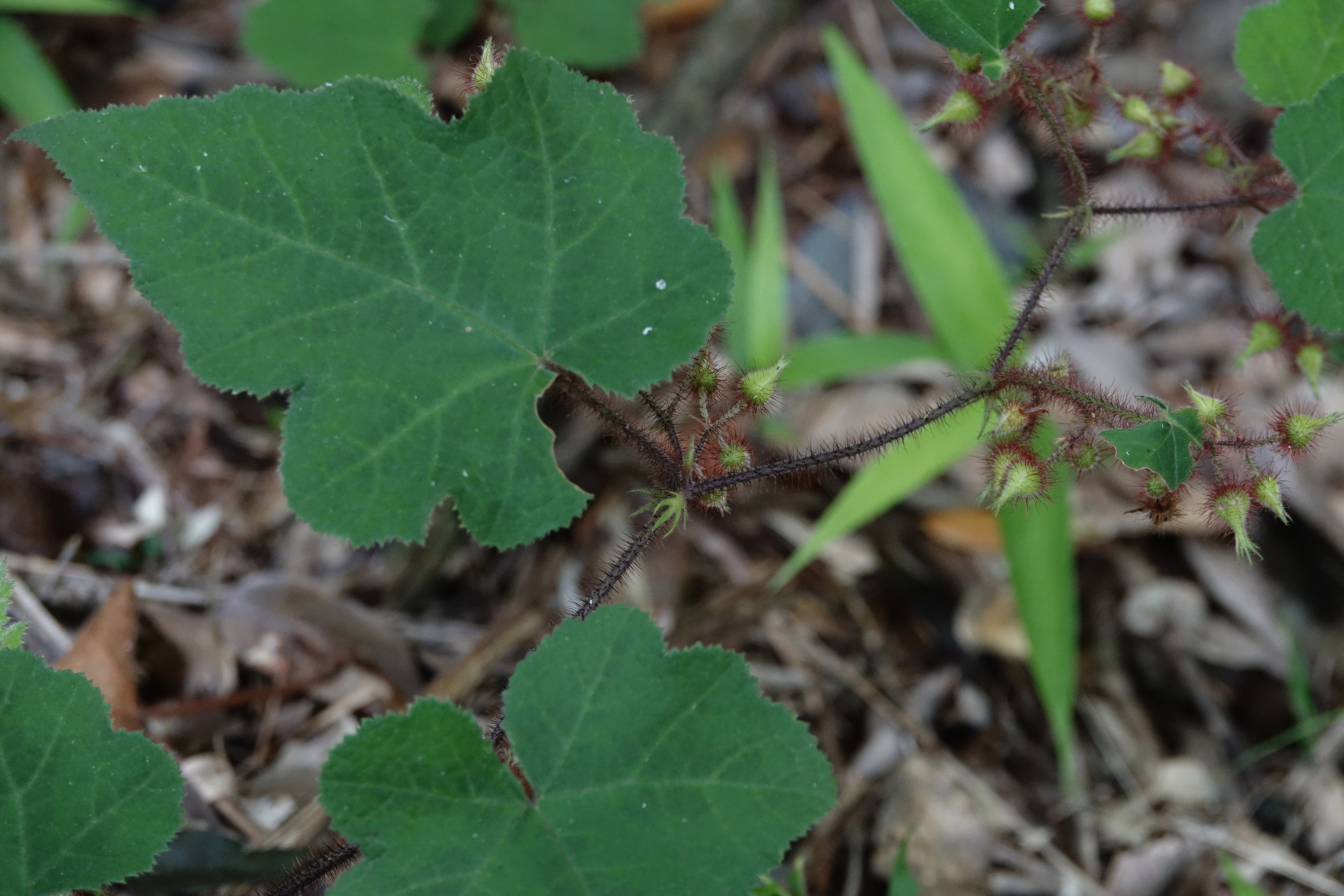

Supplement: Supplementary material 3 — Digital photos of plants R.amphidasys [file phytokeys-249-251_article-138951__-s003.zip › R_amphidasys/Rubus amphidasys_4.jpg]

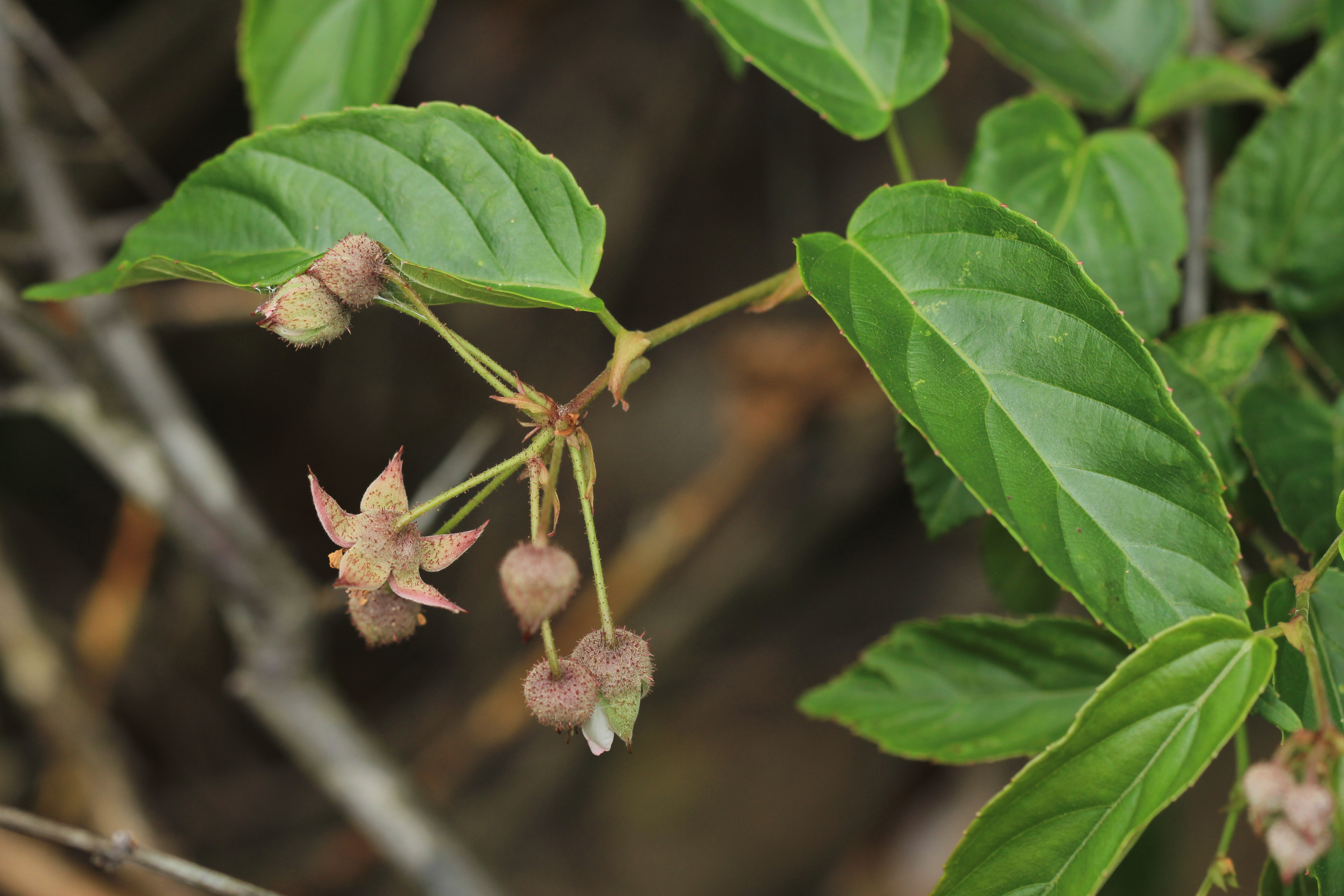

Supplement: Supplementary material 4 — Digital photos of plants R.swinhoei [file phytokeys-249-251_article-138951__-s004.zip › R_swinhoei/Rubus swinhoei_1.jpg]

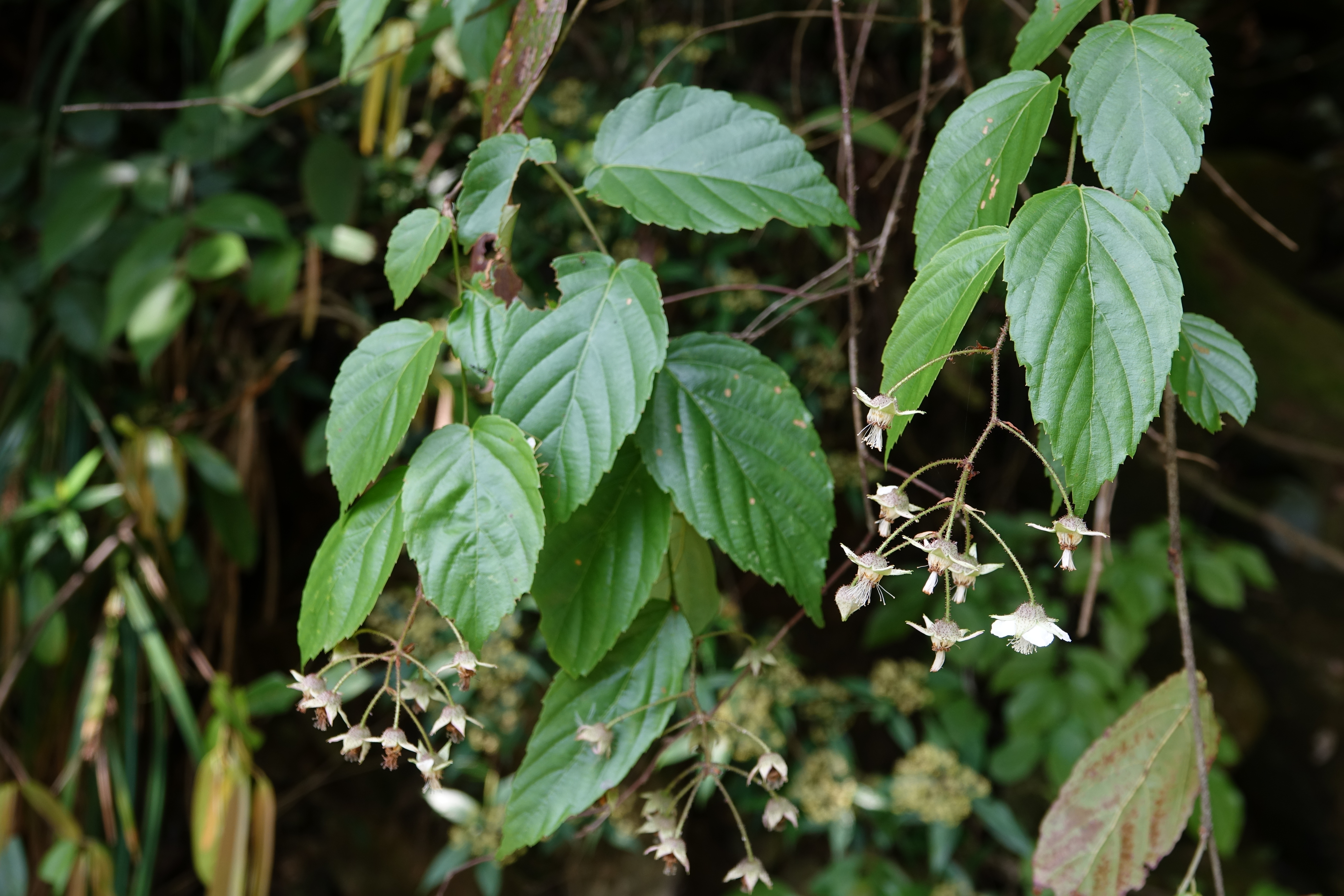

Supplement: Supplementary material 4 — Digital photos of plants R.swinhoei [file phytokeys-249-251_article-138951__-s004.zip › R_swinhoei/Rubus swinhoei_2.jpg]

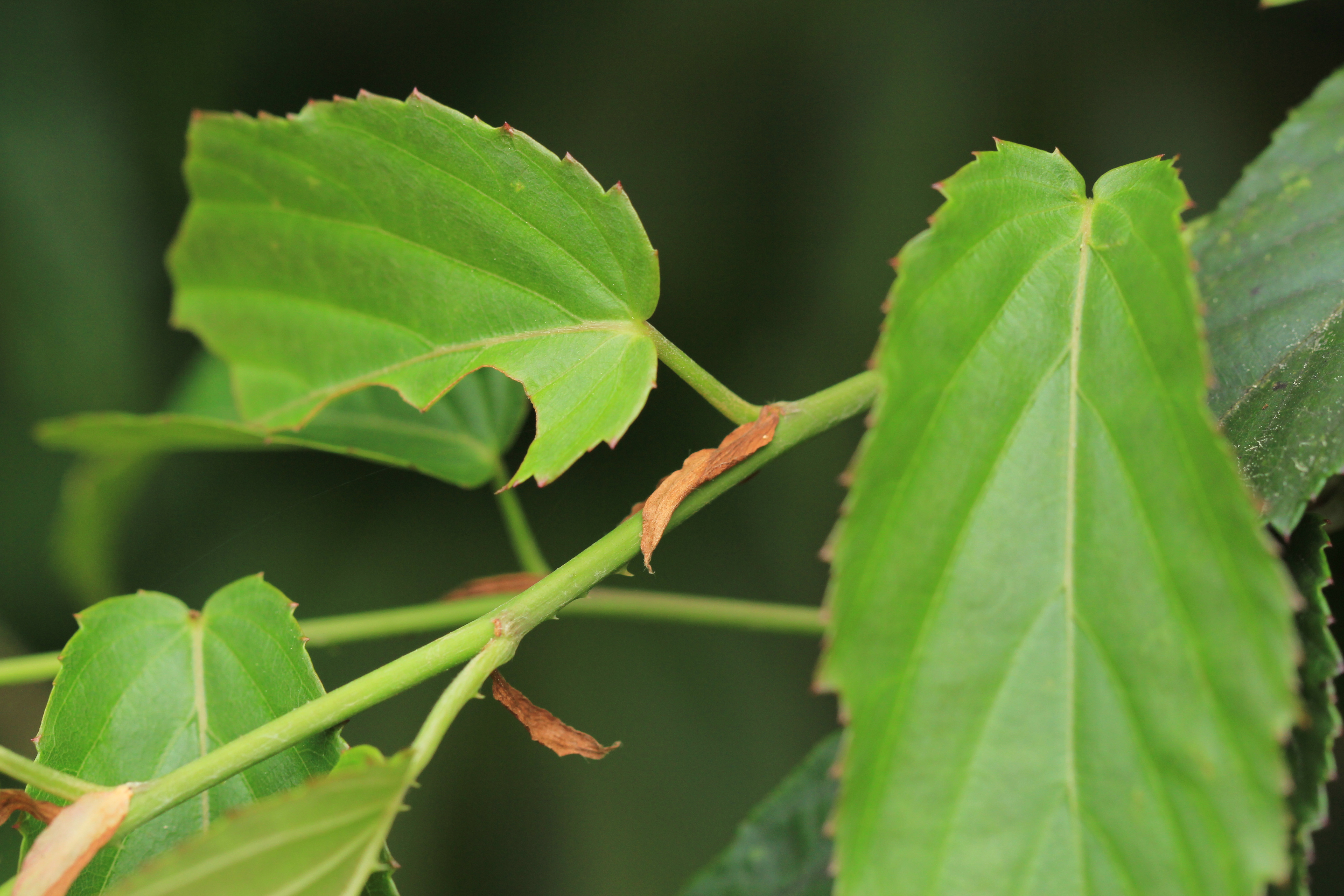

Supplement: Supplementary material 4 — Digital photos of plants R.swinhoei [file phytokeys-249-251_article-138951__-s004.zip › R_swinhoei/Rubus swinhoei_3.jpg]

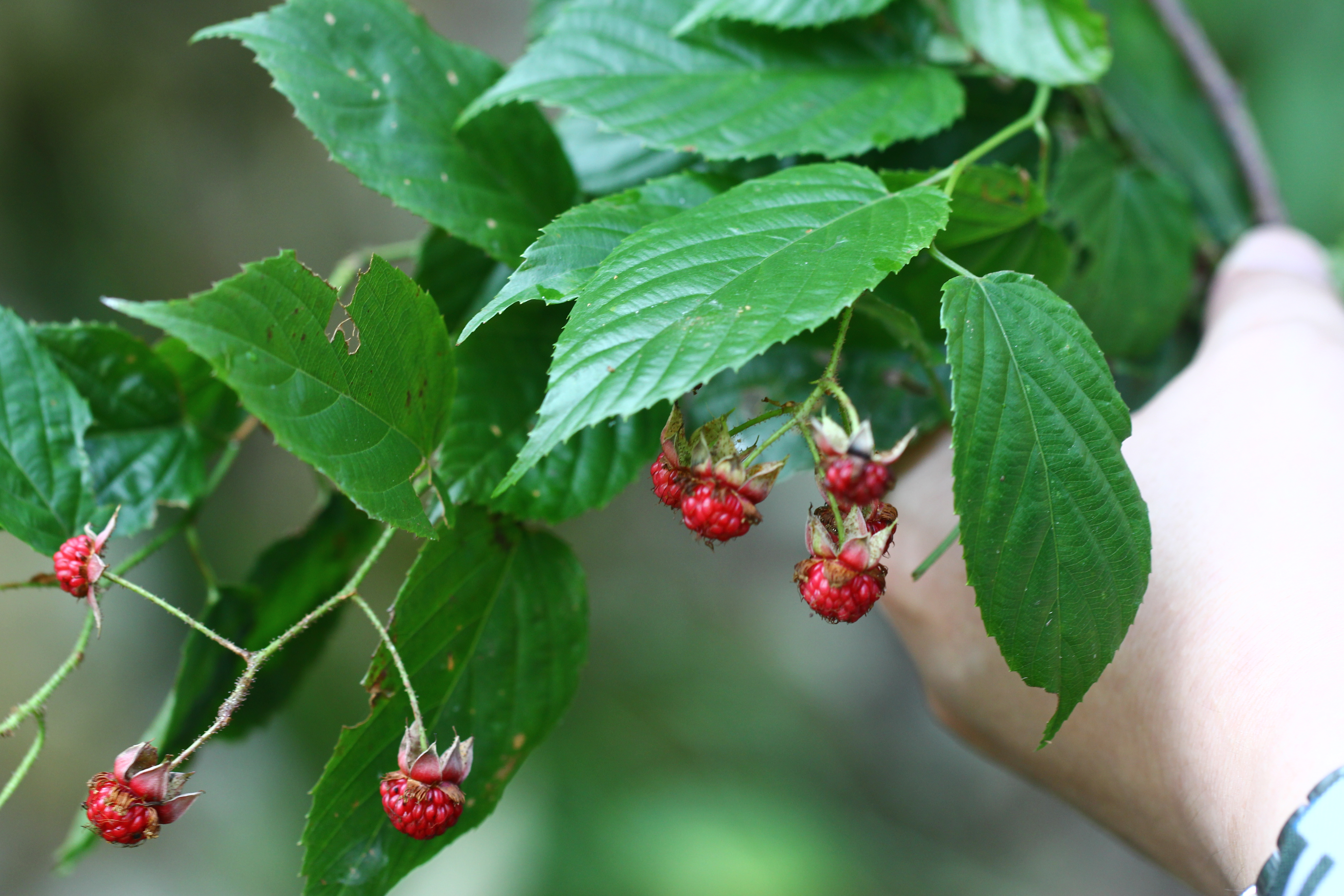

Supplement: Supplementary material 4 — Digital photos of plants R.swinhoei [file phytokeys-249-251_article-138951__-s004.zip › R_swinhoei/Rubus swinhoei_4.jpg]

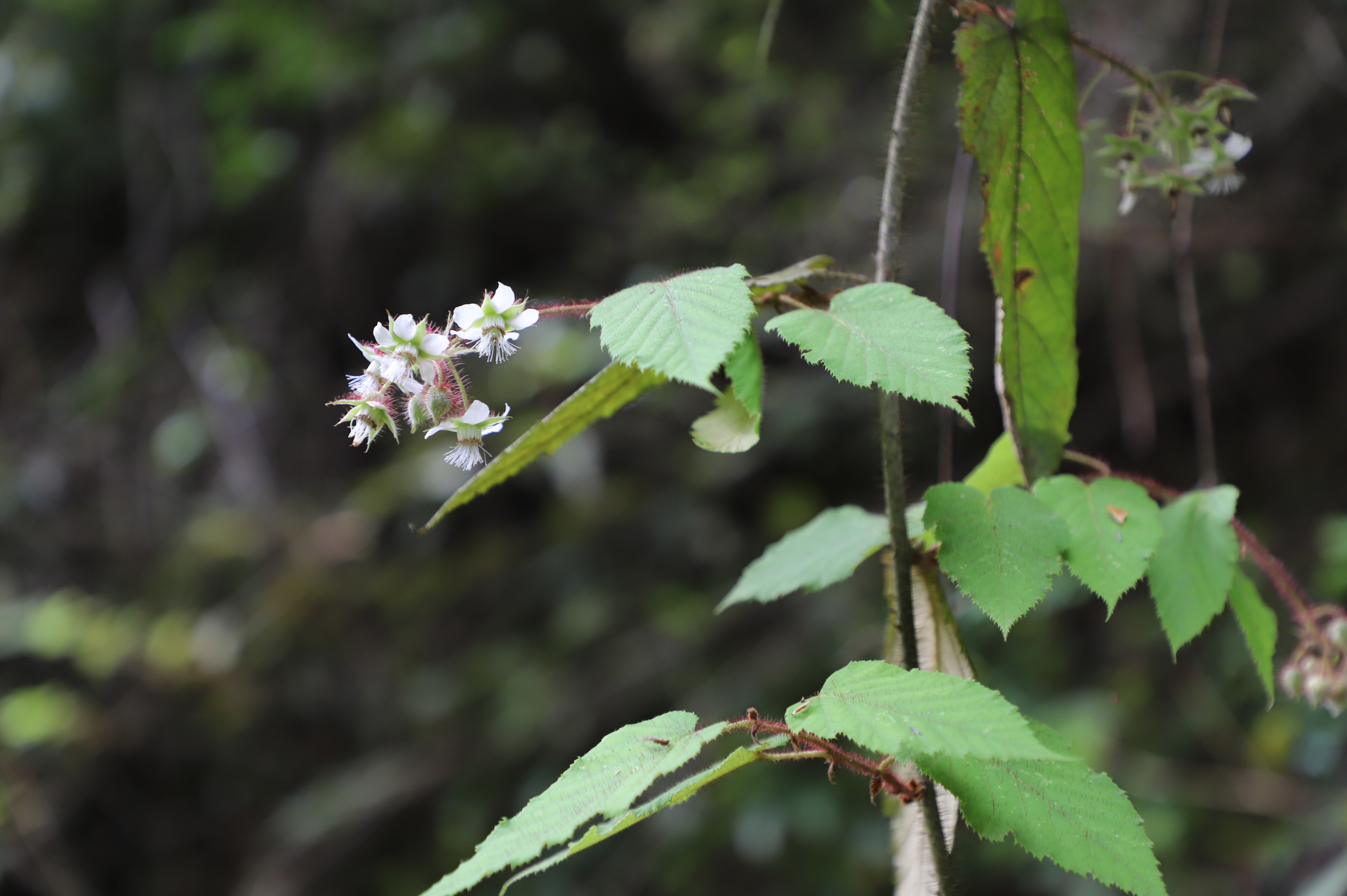

Supplement: Supplementary material 5 — Digital photos of plants R.tingzhouensis [file phytokeys-249-251_article-138951__-s005.zip › R_Changii/IMG_2757_Rubus_changii.JPG]

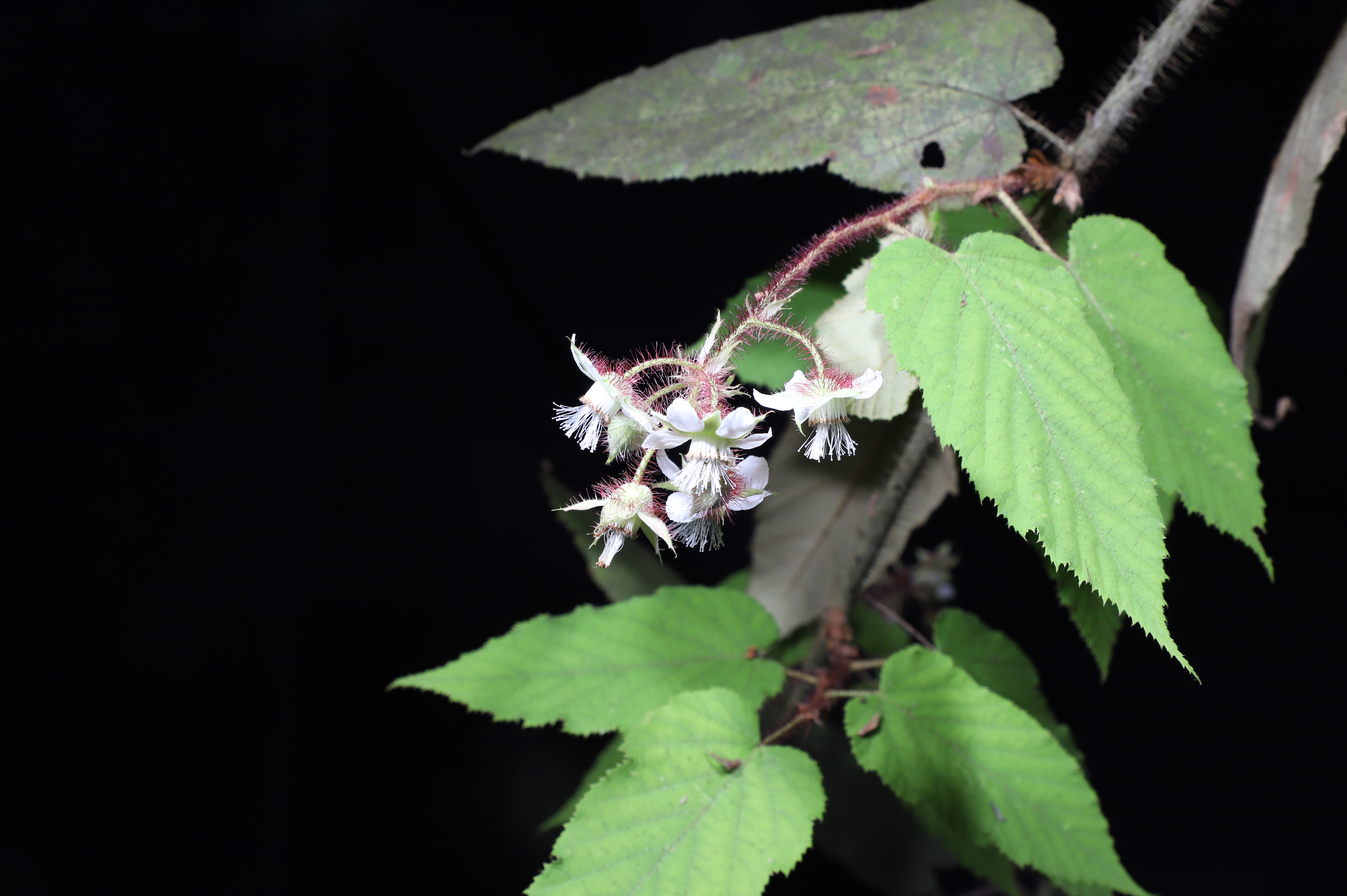

Supplement: Supplementary material 5 — Digital photos of plants R.tingzhouensis [file phytokeys-249-251_article-138951__-s005.zip › R_Changii/IMG_2775_Rubus_changii.JPG]

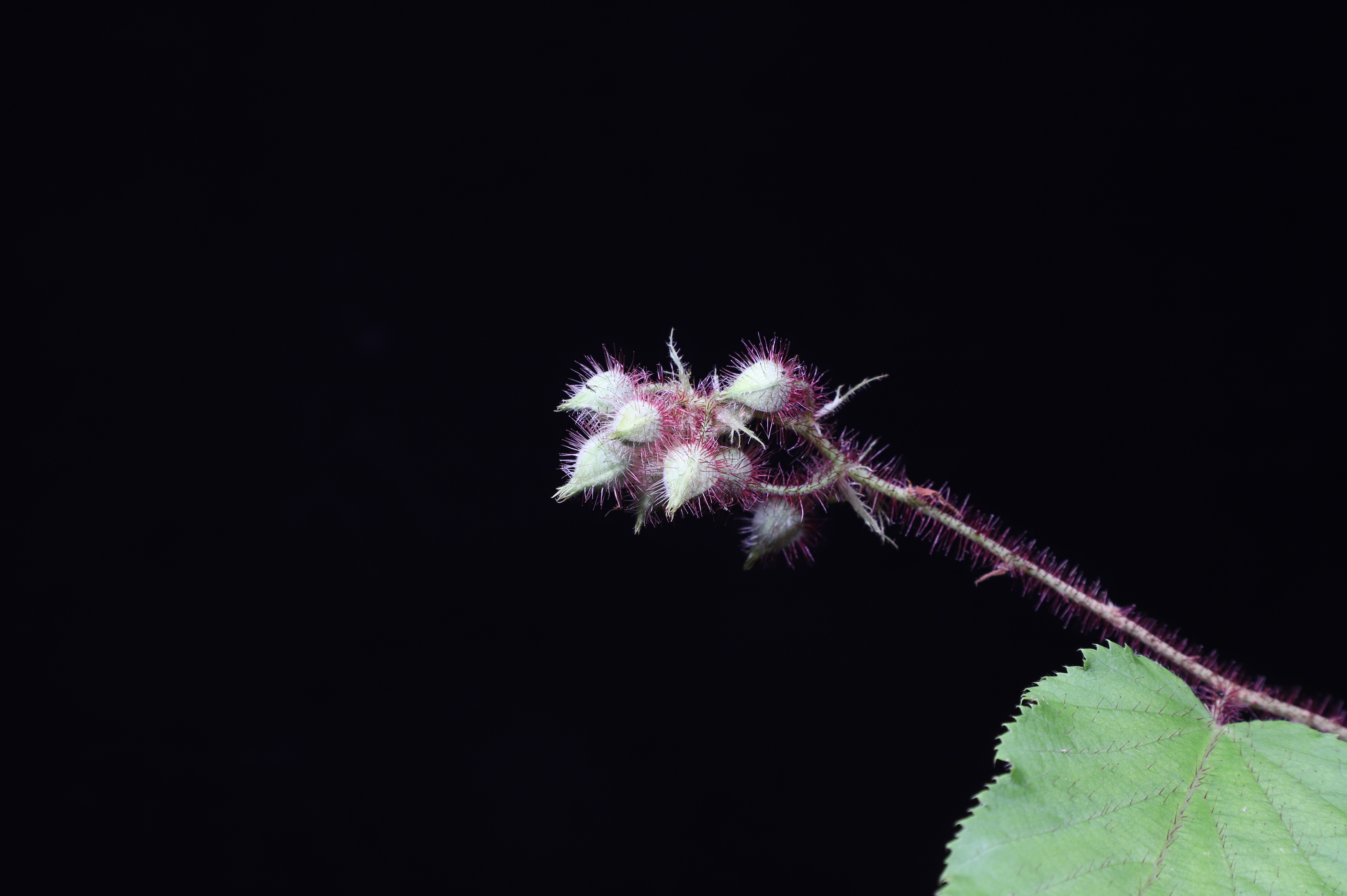

Supplement: Supplementary material 5 — Digital photos of plants R.tingzhouensis [file phytokeys-249-251_article-138951__-s005.zip › R_Changii/IMG_2789_Rubus_changii.JPG]

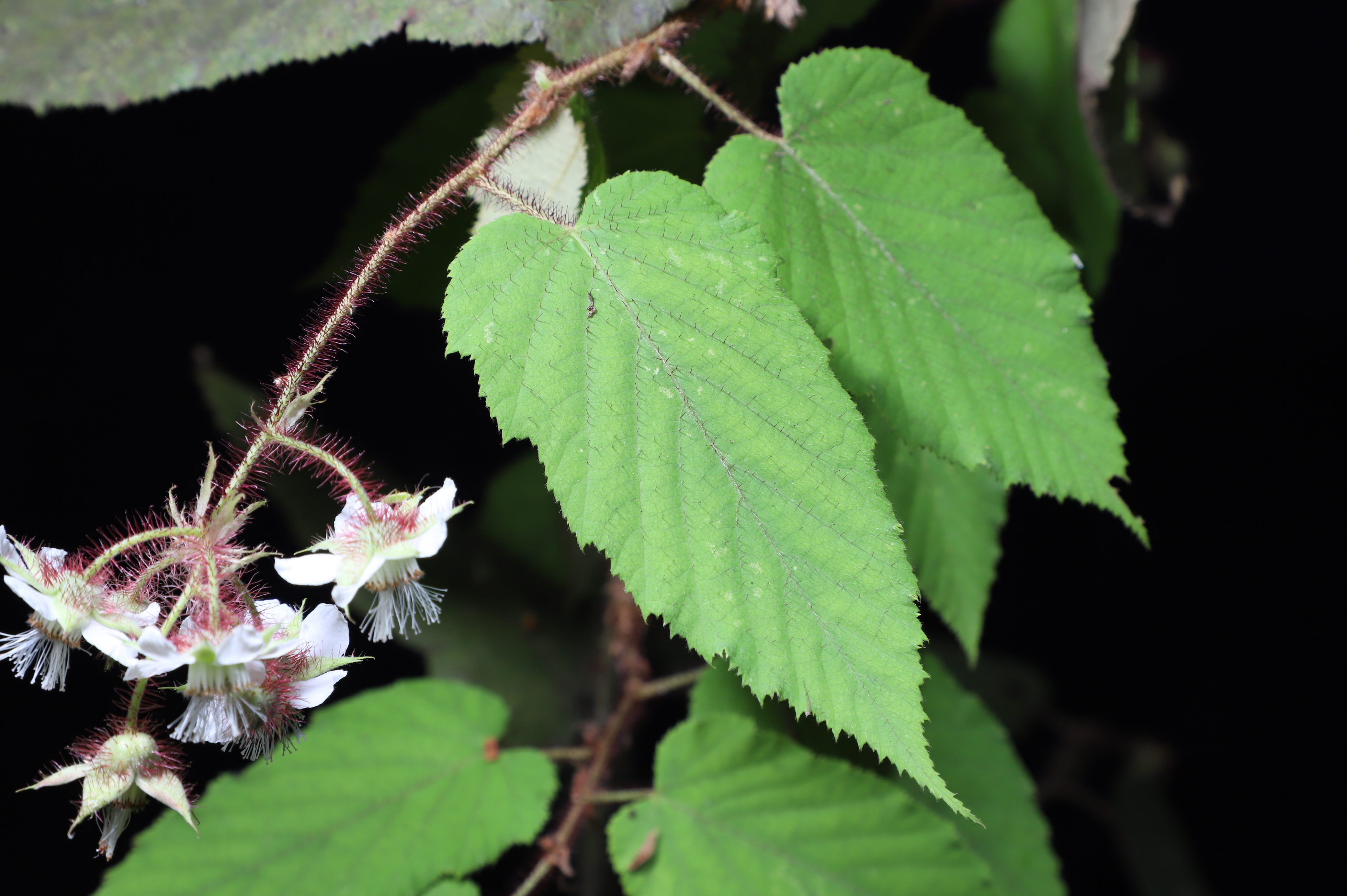

Supplement: Supplementary material 5 — Digital photos of plants R.tingzhouensis [file phytokeys-249-251_article-138951__-s005.zip › R_Changii/IMG_2796_Rubus_changii.JPG]

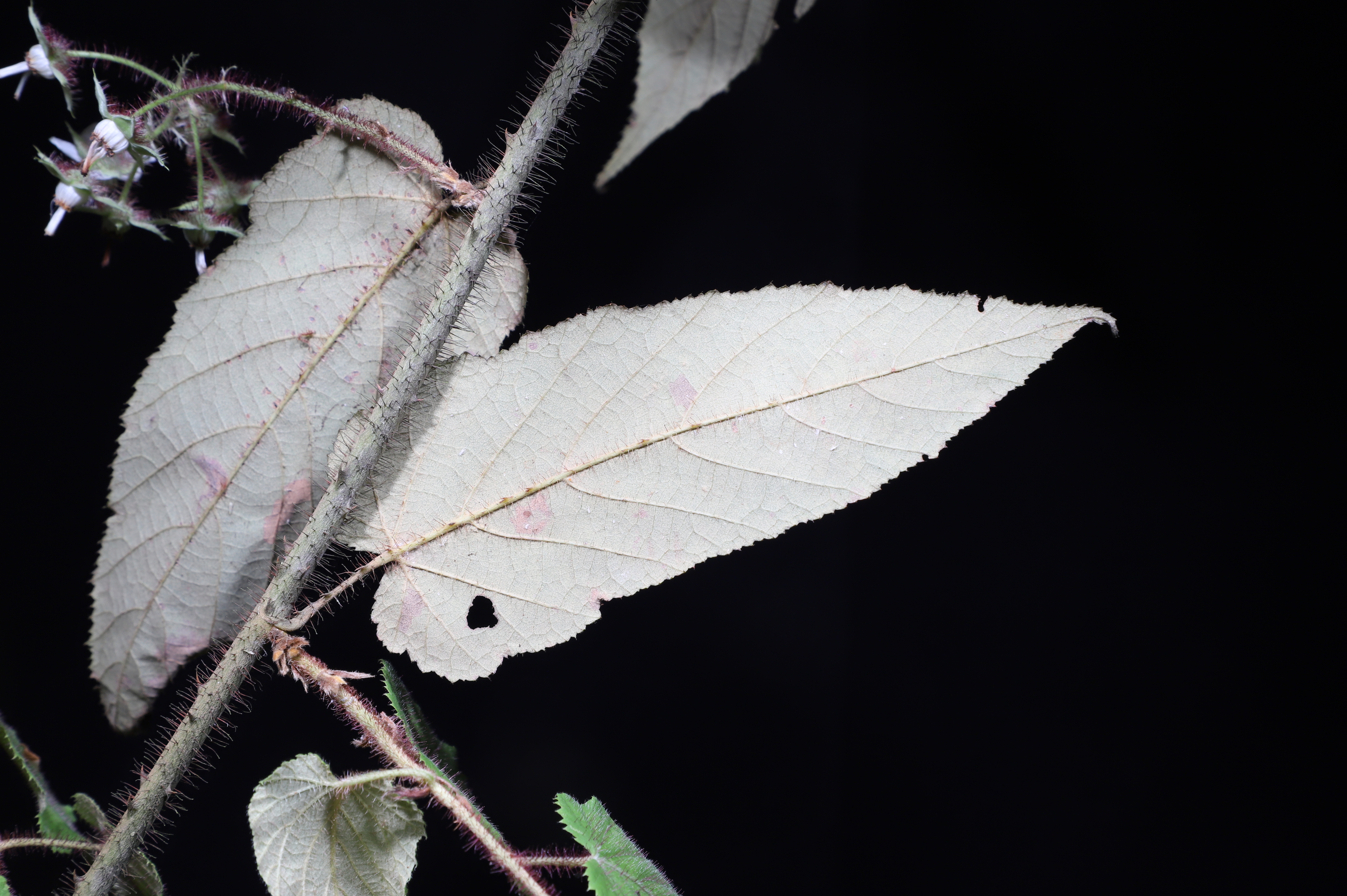

Supplement: Supplementary material 5 — Digital photos of plants R.tingzhouensis [file phytokeys-249-251_article-138951__-s005.zip › R_Changii/IMG_2797_Rubus_changii.JPG]

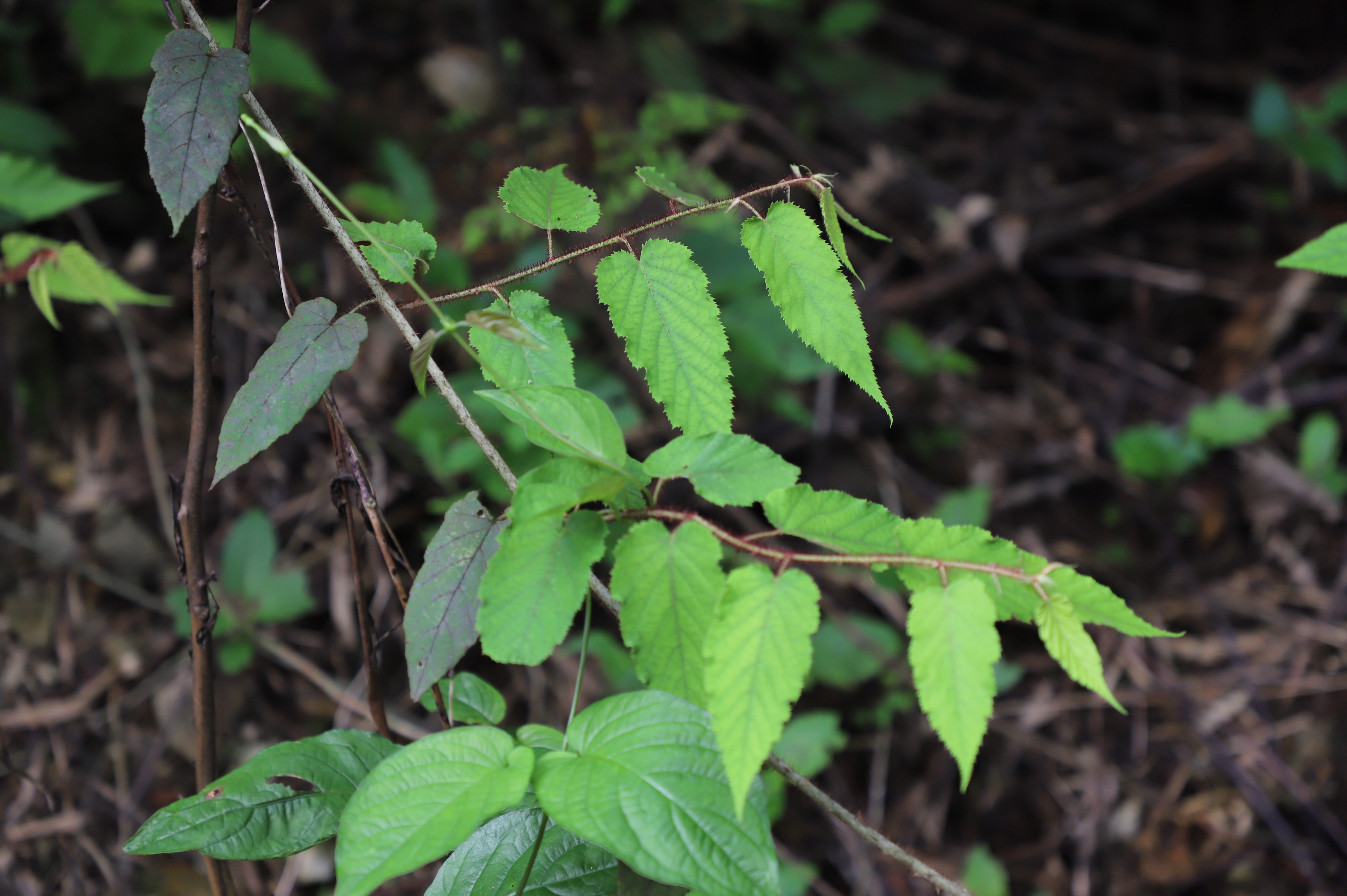

Supplement: Supplementary material 5 — Digital photos of plants R.tingzhouensis [file phytokeys-249-251_article-138951__-s005.zip › R_Changii/IMG_2813_Rubus_changii.JPG]

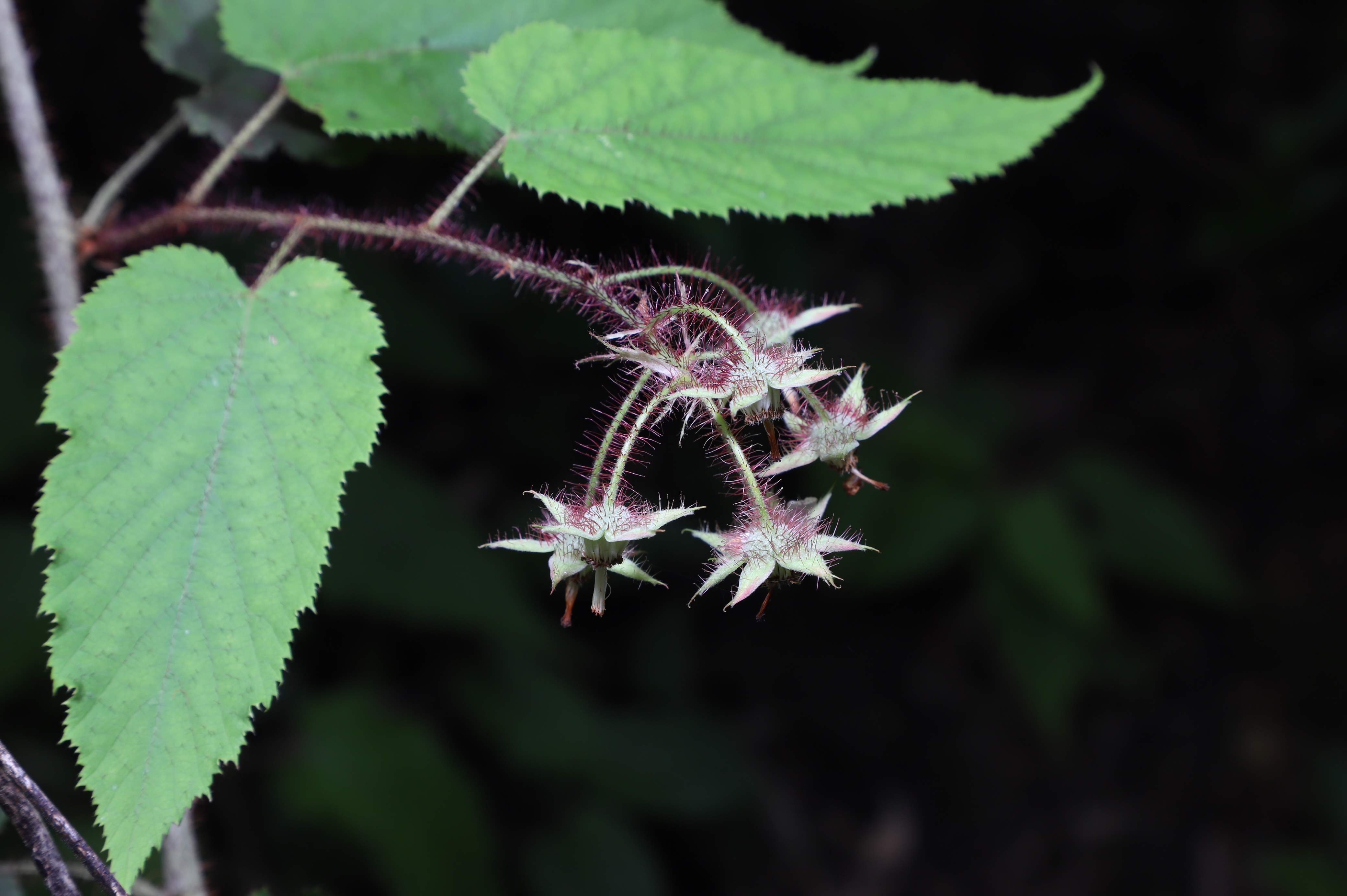

Supplement: Supplementary material 5 — Digital photos of plants R.tingzhouensis [file phytokeys-249-251_article-138951__-s005.zip › R_Changii/IMG_2819_Rubus_changii.JPG]
